# Glycans in adhesion and fertilization: histochemical and ultrastructural insights from the chaetognath *Spadella cephaloptera*

**DOI:** 10.1186/s12983-026-00614-5

**Published:** 2026-05-09

**Authors:** Cristian Camilo Barrera Grijalba, Sabine Thetter-Dürr, Julian Bibermair, Tim Wollesen

**Affiliations:** 1 Department of Evolutionary Biology, Faculty of Life Sciences, University of Vienna, Djerassiplatz 1, Vienna, 1030 Austria; 2 Research Support Facilities Imaging Unit CIUS, Faculty of Life Sciences, University of Vienna, Vienna, Austria; 3 Vienna Doctoral School of Ecology and Evolution (VDSEE), University of Vienna, Vienna, Austria

**Keywords:** Adhesive cell ultrastructure, Attachment, Chaetognatha, Gnathifera, Histology, Immunocytochemistry, Lectin affinity, Reproduction, Spiralia, Zoology

## Abstract

**Background:**

To cope with the dynamic intertidal environment, some marine invertebrates have evolved carbohydrate-based adhesive mechanisms. Glycans are also involved in reproductive processes in protostomes, yet data about their distribution and function in chaetognaths are scarce. Among them, the benthic arrow worm *Spadella cephaloptera* can rapidly and temporarily attach to substrates, making it an interesting target for investigating the underlying glycobiology of its adhesion and reproductive systems. In this study, we characterized the distribution of different glycans in *S. cephaloptera* using histochemistry, immunofluorescence, lectin binding assays, and transmission electron microscopy, with a focus on the adhesive system during ontogeny.

**Results:**

Adhesive cells showed stage-specific distribution: in hatchlings, these cells form protrusions in the epidermis (papillae) and concentrate anteriorly, while in juveniles and adults, they group into multicellular adhesive cells forming clusters (compound papillae) in the posterior region. Ultrastructurally, the adhesive cells contain secretion granules enriched in fibrillar structures, and feature synaptic vesicles. Lectin binding revealed strong Peanut Agglutinin (PNA) affinity to the apical region of the cells, indicating the presence of Galβ1-3GalNAc moieties similar to other temporary adhesive systems. In addition, we detected acidic and sulfated mucosubstances in the sperm ducts, while a carboxylated jelly coat surrounds mature oocytes.

**Conclusions:**

Our findings suggest that the ontogenetic shift of adhesive cells from the anterior to the posterior body region is correlated with the alimentary and foraging behavior during the life cycle of *S. cephaloptera*. Evidence from lectin-assays, histological stainings, and ultrastructural analyses reveals the involvement of glycans in both reproductive and adhesive systems, with patterns suggesting functional conservation of mechanisms present in other marine invertebrates. The observed glycan moieties in the adhesive cells of *S. cephaloptera* indicate convergently evolved traits, such as the presence of neutral mucosubstances and PNA-binding glycans, as reported for other temporary adhesive systems in marine invertebrates. This work provides a framework for a molecular characterization of the reproductive and adhesive systems of the enigmatic chaetognaths.

**Supplementary Information:**

The online version contains supplementary material available at 10.1186/s12983-026-00614-5.

## Background

Intertidal organisms are exposed to steep, rapidly fluctuating gradients of salinity, temperature, pH, and strong water currents [1]. Survival under these dynamic conditions requires a suite of physiological and biochemical adaptations. In this context, research on the intracellular distribution of biomolecules, such as carbohydrates, in marine invertebrates has provided insights into the unique mechanisms they have evolved [2–4]. The presence of carbohydrates in temporary attachment mechanisms has been described in a variety of marine invertebrates, including echinoderms, mollusks, chordates, and cnidarians [5–7]. Lectins are carbohydrate-binding proteins that recognize specific glycan moieties, enabling the characterization and spatial mapping of glycans in tissues. Individual lectins exhibit distinct affinities; for instance, peanut agglutinin (PNA), which binds to Galβ1-3GalNAc motifs, is strongly and specifically detected in the adhesive cells of *Macrostomum lignano*, *Ciona intestinalis*, and *Asterina gibbosa* [8–10]. Notably, in the sea star *Asterias rubens*, subsets of lectin-detected glycans have been described as part of secreted proteins, establishing specific types of glycosylation (e.g., N-/O-glycosylation) that confer electrostatic properties to the glue [2]. Additional lectins employed in the study of marine adhesive systems include wheat germ agglutinin (WGA), which recognizes N-acetylglucosamine and sialic acids, and concanavalin A (Con A), which has affinity for α-mannose and α-glucose residues [11]. However, comparable data are currently lacking for many protostomes due to comparatively limited investigations.

As both predators and prey, chaetognaths (arrow worms) play an important role in the marine food web [12–14]. They develop directly and show a mosaic of morphological and molecular traits, which has led to intense debates over their phylogenetic position [12]. Recent molecular phylogenomic analyses strongly support the placement of Chaetognatha within Gnathifera or as the sister group to the latter [15, 16]. Nevertheless, information regarding cellular processes and clade-specific innovations, including glycan-mediated processes, such as reproduction and attachment, remains scarce in chaetognaths. Understanding these processes within this phylogenetic framework is essential for interpreting chaetognath-specific adaptations.

**Fig. 1:**
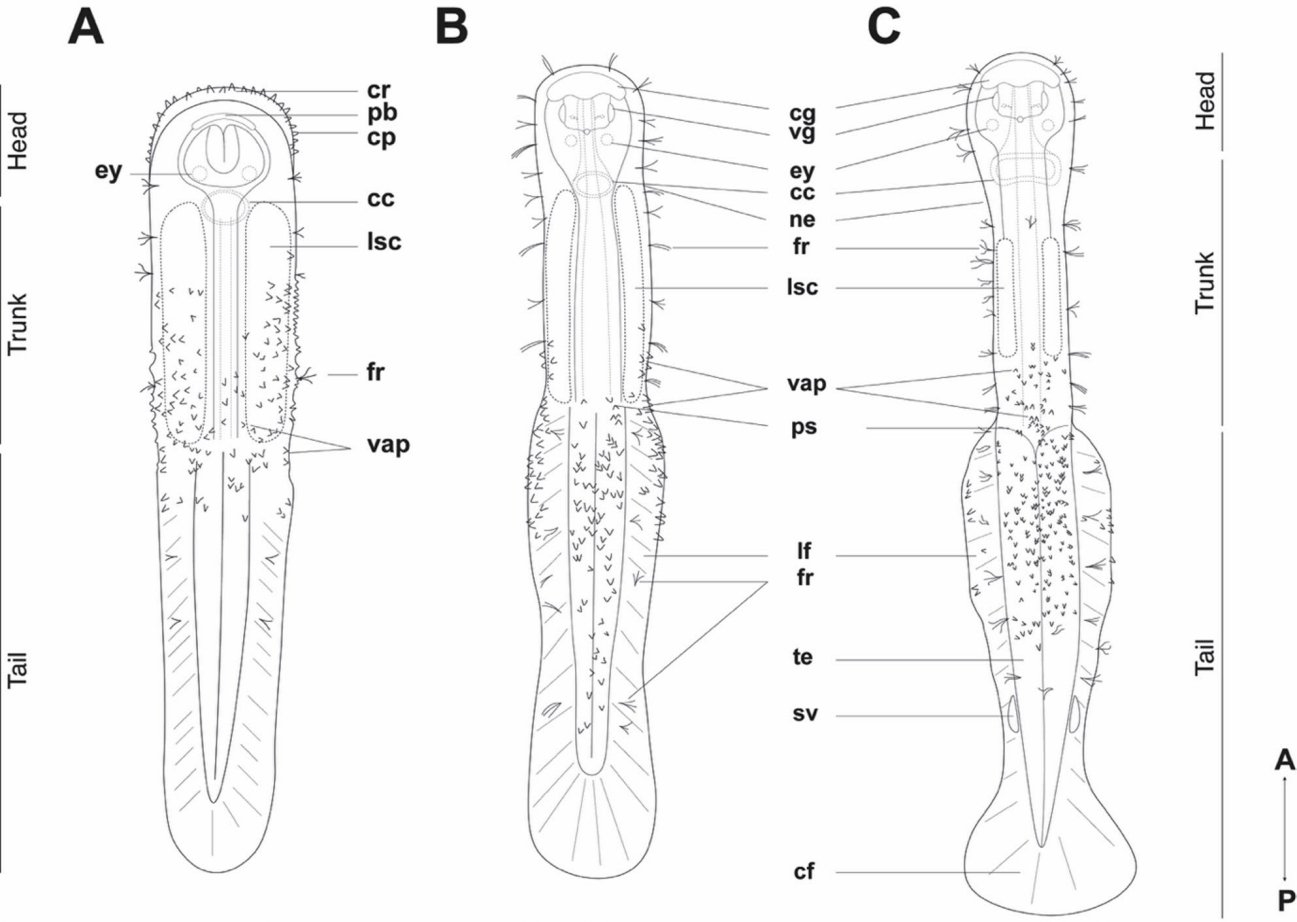
Overview of anatomical features of posthatching individuals of *Spadella cephaloptera*. The body plan features three main regions: head, trunk and tail. **(A)** Ventral view of a 24 hph specimen, exhibiting the cephalic rim (cr) in the anterior part of the head. At this stage, the feeding apparatus is not yet developed, and the animal already displays fence receptors (fr) on its epidermis. **(B)** Ventral view at 5 dph, showing further development of cephalic ganglia including the cerebral ganglion (cg) and the vestibular ganglion (vg). **(C)** Representation of the ventral view of the adult, showing a more prominent “neck” (ne) region, and reproductive system related structures, such as a pair of seminal vesicles (sv) in posterior region of the tail. Additional abbreviations: cc, corona ciliata; cf., caudal fin; cg, cerebral ganglion; cp., cephalic adhesive papillae; ey, eye; fr, fence receptors; lf, lateral fin; lsc, lateral somata cluster; pb, primordial brain; ps, posterior septum; vap, ventral adhesive papillae

As the best-studied chaetognath, the benthic species *Spadella cephaloptera* exhibits a temporary adhesive system characterized by clusters of adhesive cells that form epidermal protrusions (papillae) in the posterior body region and are present throughout development (Fig. 1) [17–21]. These cells display electron-dense secretory granules in the apical region when examined by transmission electron microscopy (TEM) [12], yet their molecular composition remains unknown.

Analyzing the glycan composition of the adhesive system of *S. cephaloptera* offers the possibility to identify commonalities and differences in wet adhesion in a broader range of taxa. Secretions from marine invertebrates have already inspired solutions in hydrogel formation, wet glues, and insights for biofouling prevention [6, 22]. For example, extensive research has been performed on bivalve byssus—long, silky threads secreted by pedal glands for substrate attachment. In *Mytilus edulis*, the role of 3,4-dihydroxyphenyl-l-alanine (DOPA) has been well characterized. DOPA is a modified amino acid with adhesive properties associated with a catechol group that favors interactions such as hydrogen and metal-ion coordination and cationic interactions [23]. DOPA has been utilized as the core of multiple bio-inspired applications, providing a route for designing materials that mimic its chemical properties in the search for a product with the original properties of bivalve glue [24]. While the properties of DOPA are relevant for the attachment process, some other players, including glycans, remain comparatively understudied [25, 26]. Carbohydrates are known to provide hydrophilic properties, facilitate hydrogen bond formation and contribute to the viscoelasticity properties in various adhesive systems [2].

In addition to bioadhesion, glycans also play essential roles during the fertilization process in the sea urchin *Strongylocentrotus purpuratus*, mediating gamete recognition and triggering downstream mechanisms such as the release of cortical secretory granules to prevent polyspermy [27, 28]. In the chaetognath *Parasagitta elegans*, fluctuations in whole-body lipid and carbohydrate levels coincide with the reproductive cycles [29], but the roles of polysaccharides during this process remain unknown. Chaetognaths are protandric hermaphrodites, and spadellid species in particular are known for their elaborate mating behavior [30]. While ultrastructural studies of the fertilization process in *S. cephaloptera* exist, information on the glycan composition of sexual gametes is lacking [12, 31]. Understanding chaetognath glycobiology is particularly relevant, as environmental factors, such as pH, affect fertilization success in marine invertebrates. The latter includes, for example, successful interactions between the oocytes and sperm and their decline in acidic conditions [32].

We investigated glycan distribution in the adhesive and reproductive systems of *S. cephaloptera* using histochemical, immunohistochemical, and ultrastructural analyses. Our results indicate an epithelial secretion mechanism for adhesion, notably lacking a dual-gland system. Adhesive cells transition from individual cells during early stages to distinct clusters in adulthood. While there are no direct neural connections between ganglia and adhesive cells, the distribution of acetylated α-tubulin filaments, alongside the presence of synaptic vesicles within the adhesive cells, suggests a relationship between the adhesive system and an intraepidermal nervous plexus. Similar to other marine invertebrates, Galβ1-3GalNAc glycoconjugates, as revealed by the lectin peanut agglutinin (PNA), are present in the apical region of the chaetognath adhesive cells [9, 10, 33]. Additionally, we show that sulfated and carboxylated polysaccharides in the seminal fluid of the testis ducts may provide metabolic support for sperm cells. In contrast, exclusively carboxylated polysaccharides in mature oocytes suggest a role for carbohydrate-mediated gamete recognition.

## Methods

### Animal collection and husbandry

Adult specimens of *Spadella cephaloptera* were collected at low tides using plankton nets on the shore adjacent to the Station Biologique de Roscoff (Roscoff, France), at the approximate location: 48°43’47.2"N, 3°59’15.3"W, in the summer of 2023. The specimens were placed in Petri dishes with natural seawater at 35 ppt to induce mating and egg laying. Egg batches were transferred to separate petri dishes to record individual hatching times, and seawater was exchanged daily. Before fixation, all the studied specimens from the different stages of *S. cephaloptera* were relaxed in a solution containing 100 mM MgCl2 in filtered seawater and incubated at 4 °C depending on their developmental stage: 10 min for 24 h post-hatching (24 hph) and 5 days post-hatching (5 dph) old specimens (time points defined since the hatchling leaves the egg); 2 h for late juveniles and adults.

### Transmission electron microscopy

Specimens at 24 hph, 5 dph, and adults of *S. cephaloptera* were prepared according to established protocols with modifications [18]. The samples were fixed in Karnovsky’s solution for 2 h in a water bath at 48 °C (3% glutaraldehyde, 1% paraformaldehyde, and 30% filtered seawater containing 0.2 M sodium cacodylate buffer, pH 7.3). Afterward, the specimens were rinsed every 30 min for 2 h using the cacodylate solution containing 9% glucose. Post-fixation was done in 1% osmium tetroxide in distilled water for 1 h at room temperature, followed by repeated rinsing and finally dehydration in an ascending ethanol series and 100% acetone. Later, the samples were gradually infiltrated with epoxy resin, Agar 100 (Agar Scientific), overnight on an orbital shaker at room temperature, using acetone as an intermediate, embedded, and polymerized at 65 °C for 70 h. Subsequently, ribbons of 1 μm serial sections were produced using a Histo Jumbo diamond knife (Diatome) on a Leica Ultramicrotome UC6 (Leica Microsystems), as previously reported [34]. The generated sections were stained with a 1% Toluidine Blue solution for 25 s at 80 °C. Stained sections were analyzed using a light microscope, and sections containing a region of interest were resectioned to 60–80 nm following established protocols [35]. Ultra-thin sections were contrasted using 1% uranyl acetate for 8 min, followed by three rinses. Next, the grids were counterstained for 3 min in 3% lead citrate and rinsed thrice again. Ultrastructure of the ultra-thin sections was documented using a ZEISS Libra 120 transmission electron microscope (Zeiss). The resulting images were later processed using Fiji v. 2.16.0 [36] and IrfanView v. 4.72 [37].

### 3D reconstruction

The semi-thin serial sections of 24 hph and 5 dph specimens of *S. cephaloptera* were used for 3D reconstruction of the ventral epidermal papillae. The sections were imaged using a Nikon DS-Ri2 camera mounted on a Nikon Ni-U compound microscope (Nikon). The image stack was loaded into Fiji v.2.16.0 [36], where it was transformed into 8-bit greyscale images. Images unsuitable for reconstruction were deleted and replaced with adjoining images, using the stack sorter tool of Fiji. The resulting stack was registered in Amira v. 2022 (Thermo Scientific) and aligned using the AlignSlices module with manual adjustments. Adhesive cells identified in the epidermal surface of *S. cephaloptera* were manually segmented in the Segmentation Editor. The surface of the adhesive papillae was generated following the steps of previous studies [38]. The reconstructed cells were visualized in the specimen via a volume rendering of the latter combined with a surface viewer module. Snapshots were exported for further image processing.

### Histological staining procedures

Adult specimens of *S. cephaloptera* were fixed for one hour in Bouin’s solution (72% picric acid, 4% formaldehyde, 24% glacial acetic acid) at room temperature. Later, the samples were washed four times for 15 min each with 70% ethanol and stored at 4 °C before further processing. The samples were dehydrated in an ascending ethanol series. Next, the specimens were transferred into benzol for 1 h and finally incubated in paraffin at 60 °C overnight. Subsequently, paraffin blocks were stored at 4 °C for 16 h before sectioning. Ribbons of thin sections of 6 μm were produced using the Leica RM2235 microtome (Leica) and later stained with the following staining procedures to identify the general properties of the glycans present in the tissue: Alcian Blue (pH = 2.5) for detecting carboxylated and sulfated polysaccharides; Alcian Blue (pH = 1.0) for targeting exclusively sulfated polysaccharides, both with hematoxylin counterstain, and Periodic Acid-Schiff reaction (PAS), for detecting neutral glycans [39–41]. To evaluate the contribution of glycogen to PAS reactivity, intercalated sections were pre-treated with α-amylase (type VI-A from porcine pancreas, Sigma-Aldrich; 1 mg/ml in phosphate-buffered saline, pH 7.0) for 15 min at 37 °C prior to the PAS staining protocol. PAS-positive staining that persists after the α-amylase treatment indicates other neutral carbohydrates different from glycogen [40]. Stained slides were sealed with DMX and documented using a Nikon DS-Ri2 camera mounted on a Nikon NI-U microscope.

### Immunohistochemistry

Hatchlings, juveniles, and adults were fixed overnight at 4 °C in a fixing solution (4% paraformaldehyde, 0.1 M MOPS, 2 mM MgSO_4_, 1 mM EGTA, 0.5 M NaCl). The samples were washed three times for 5 min with PBS-T (1X PBS, pH 7.4, and 0.01% Tween 20) and then stored in PBS with 0.05% Sodium Azide. Afterward, the specimens were incubated in PBTr (1x PBS pH 7.4 with 0.3% Triton X-100(v/v)) and washed three times for 10 min. From this point onwards, and up to sample mounting, incubations were performed at 4 °C. Non-specific antibody binding was blocked using PBTr supplemented with 3% (v/v) Normal Goat Serum, NGS (Invitrogen), on a rotatory shaker at 70 rpm overnight. Subsequently, the samples were incubated in the primary antibody (anti-acetylated α-tubulin from mouse) (Sigma) diluted 1:2000 in the blocking solution overnight. The unbound primary antibody was washed three times with PBTr for 10, 30, and 60 min. Later, the samples were incubated overnight in a secondary antibody (anti-mouse Alexa 633) (Thermo Fischer Scientific) at 1:500 in PBTr supplemented with 1% NGS. The excess antibody was removed with three PBTr washes for 60 min. At this point, the samples were gradually transferred to PBS-T (1x PBS, pH 7.4, with 0.05% Tween 20) and washed three times with the same solution for 10 min. Subsequently, to stain F-actin filaments, the samples were incubated overnight in PBS-T supplemented with Alexa Fluor^®^ 488 phalloidin (Invitrogen) at a dilution of 1:40 and (0.3%) DAPI in the dark. The excess phalloidin and DAPI (Carl Roth) was removed using PBS-T washes, repeated 8 times for 10 min each. Next, the specimens were mounted on glass slides with Vectashield (Vector Laboratories) and visualized using a Leica Stellaris 5/DMi8 confocal microscope (Leica) with sequential fluorescence scans.

### Lectin affinity assay

Hatchlings (24 hph) of *S. cephaloptera* were fixed as described for the immunohistochemistry assays. Lectin fluorescence histochemistry was performed as previously described with modifications [10]. Briefly, the specimens were washed four times for 15 min in TBS-T (TRIS buffer, pH 8.0, supplemented with 5 mM CaCl_2_ and 0.1% Triton X-100). From this step onward, all incubations were carried out at 4 °C. Non-specific binding was blocked with TBS-T containing 3% (w/v) bovine serum albumin (Sigma-Aldrich) overnight. Subsequently, biotinylated lectins (Vector Laboratories) were added at a final concentration of 25 µg/ml and incubated overnight. Later, unbound lectins were washed using TBS-T six times for 10 min. To fluorescently label the attached lectins, the samples were incubated for 1 h in the blocking solution with a 1:300 dilution of streptavidin conjugated to Alexa Fluor 633 (Sigma-Aldrich). The samples were washed eight times for 15 min with TBS-T and then incubated for 1 h with DAPI at a ratio of 1:350 in TBS-T. After removing the excess stain with three washes of TBST-T for 5 min each, the samples were mounted in Vectashield and visualized using a Leica Stellaris 5 / DMi8 - confocal microscope (Leica). For each lectin, we carried out three independent staining experiments using several 24 hph old hatchlings.

## Results

### Carbohydrate distribution in the adult chaetognath *Spadella cephaloptera*

**Fig. 2:**
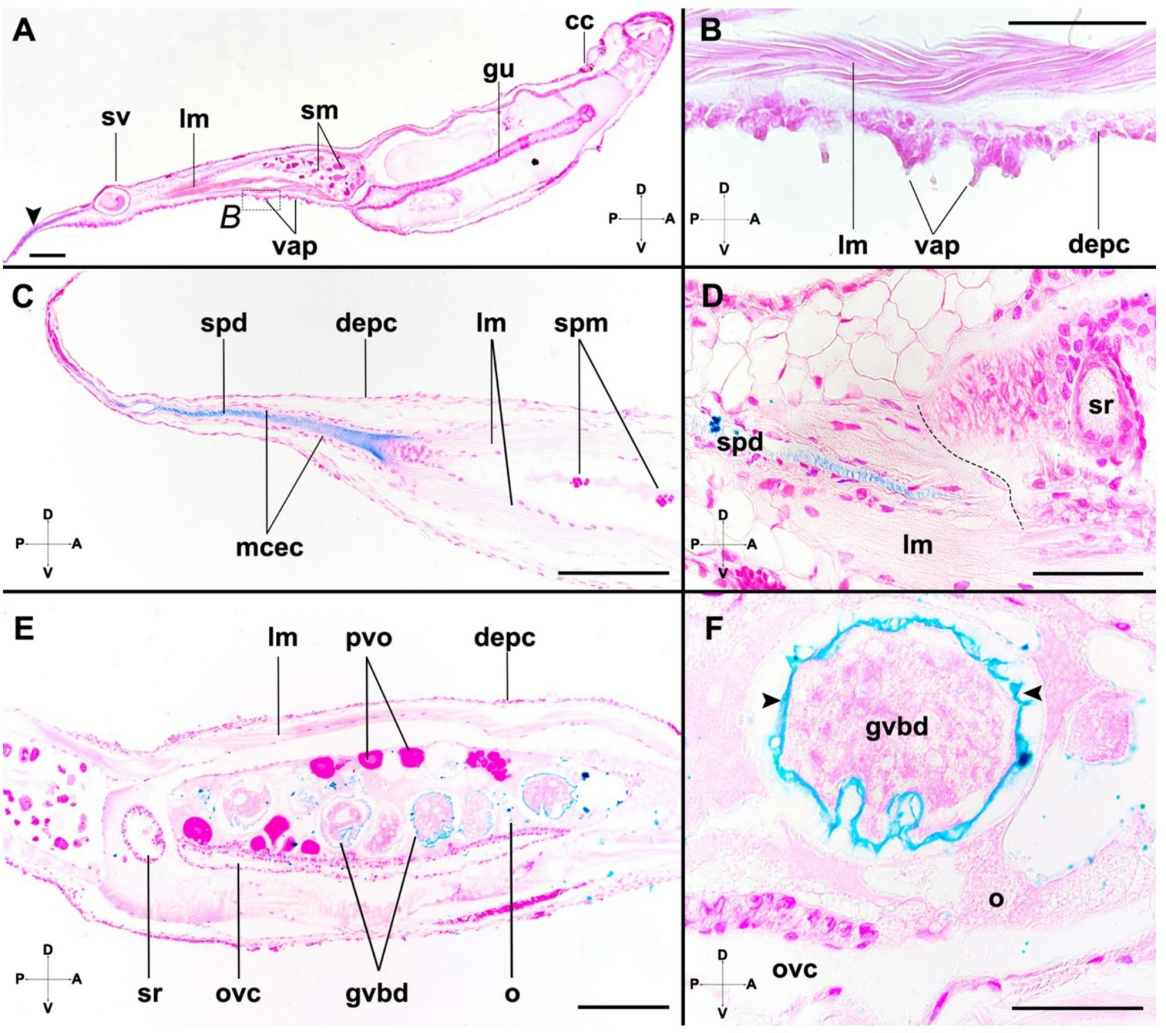
Location of carboxylated and sulfated polysaccharides in the adult chaetognath *Spadella cephaloptera*. The cell nuclei are counterstained with hematoxylin (magenta) and the polysaccharides are stained with Alcian blue pH 2.5 (blue). **A**. Sagittal section displaying the overview of the coelomic cavities and internal organs and structures of *S. cephaloptera*. Stained polysaccharides are visualized in the posterior end of the tail (arrowhead), near the seminal vesicles (sv). **(B)** Detailed aspect visualizing the ventral adhesive papillae (vap) that are surrounded by distal epidermal cells (depc) but devoid of evident staining related to the presence of detectable polysaccharides at pH 2.5. **(C)** Close-up in the posterior end of the tail, where the sperm duct (spd) is enriched in sulfated and carboxylated polysaccharides. **(D)** Limit between the tail coelom and the trunk cavities, displaying staining of Alcian blue up to the posterior septum (dotted line). **(E)** Overview of the distribution of sulfated and carboxylated polysaccharides in the trunk cavities, which contain mature oocytes after the germinal vesicle breakdown (gvbd). Pre-vitellogenic oocytes (pvo) do not feature these glycans. **(F)** Detail of one mature oocyte contained in the ovary exhibiting carboxylated and sulfated polysaccharides in the presumptive jelly coat (arrowheads). Additional abbreviations: gu, gut; cc, corona ciliata; lm, longitudinal muscles; mcec, multiciliated epithelial cells; o, ovary; ovc, oviducal complex; sm, spermatogonial masses; sr, seminal receptacles. Scale bars: A = 200 μm; B, D, F = 50 μm; C, E = 100 μm

The distribution of acidic or carboxylated polysaccharides detected by Alcian Blue (pH = 2.5) is found in distinctive reproductive structures but not in the adhesive cells in adults of *Spadella cephaloptera* (Fig. 2A, B). The sperm ducts, located between the longitudinal muscles of the tail, but not the seminal vesicles, contain acidic or carboxylated polysaccharides (Fig. 2A, C). The distribution of acidic or carboxylated polysaccharides in the sperm ducts extends to the posterior septum, an internal partition that separates the body cavities into the trunk and the tail, encompassing the entire length of the duct (Fig. 2D). In the trunk region, the mature oocytes reside within the coelomic cavities surrounded by an Alcian Blue^+^ (pH = 2.5) coat covering the ovulated eggs [29]. The eggs move afterwards into the oviducal complex as part of the trunk cavities (Fig. 2E, F).

**Fig. 3:**
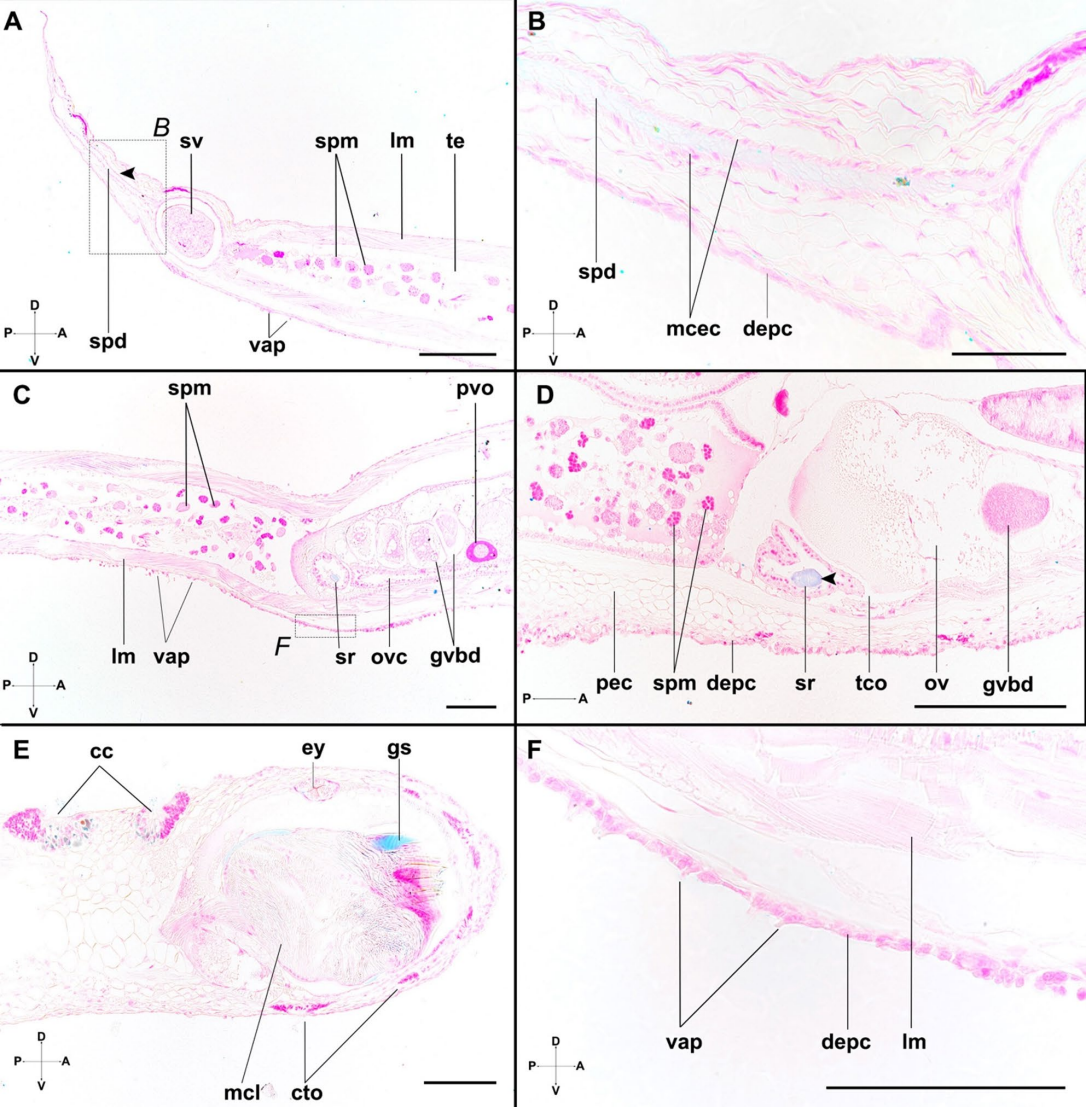
Distribution of sulfated polysaccharides in the adult chaetognath *Spadella cephaloptera*. The cell nuclei are stained with hematoxylin (pink/magenta), and the glycans are visualized with Alcian blue pH 1 (blue/ cyan). **(A)** Sagittal section of the posterior end of the tail, in which the sperm duct (spd) exhibits the presence of sulfated polysaccharides. **(B)** Detail of the sperm duct (referenced in A), which is surrounded by multiciliated epithelial cells (mcec). **(C)** Overview of the transition between trunk and tail, where there is staining located in the seminal receptacle (sr), and there is no evidence of sulfated polysaccharides in the jelly coat of oocytes after the germinal vesicle breakdown (gvbd), nor in the pre-vitellogenic oocytes (pvo). **(D)** Frontal section showing a close-up of the seminal receptacle (sr), which contains a defined vesicle with staining for sulfated glycans (arrowhead). **(E)** Sagittal section of the anterior region of the head of the adult chaetognath, where the grasping spines around the vestibular musculature exhibit chitinous structures that are detected by Alcian blue pH 1. Notably, there is also staining derived from the ciliary structures of the corona ciliata (cc). **(F)** Close-up of the ventral adhesive papillae (vap) from the tail (referenced in C), for which there is no evidence of the presence of acidic mucosubstances. Additional abbreviations: cc, corona ciliata; cto, ciliary tuft organs; depc, distal epidermal cells; ey, eye; gs, grasping spine; lm, longitudinal muscle; mcl, lateral muscle; ov, ovary, ovc, oviducal complex; pec, proximal epidermal cells; spm, spermatogonial masses; sv, seminal vesicle; tco, trunk coelom; te, testis. Scale bars: A = 200 μm; B, F = 50 μm; C, D-E = 100 μm

The location of the exclusively sulfated polysaccharides, as detected by Alcian Blue (pH = 1.0), overlaps with the staining patterns observed at pH 2.5 (cf. Figures 2 and 3). In this regard, the staining in the tail coelom is visible in the sperm ducts at a lower intensity; however, it was not observed inside the sperm vesicles (Fig. 3A-B). In contrast, in the trunk region, acidic polysaccharides were not detected covering the mature oocytes (Fig. 3C). Staining was, however, observed inside the seminal receptacles, which occur bilaterally above the posterior septum (Fig. 3C-D). In the head region, the chitinous grasping spines that insert at the lateral plates exhibited AB^+^ (pH = 1.0) staining (Fig. 3E). Furthermore, the ventral adhesive papillae showed no detectable sulfated mucosubstances, which suggests that the molecules inside the secretion granules may display different glycan moieties (Fig. 3F; cf. [12]).

**Fig. 4:**
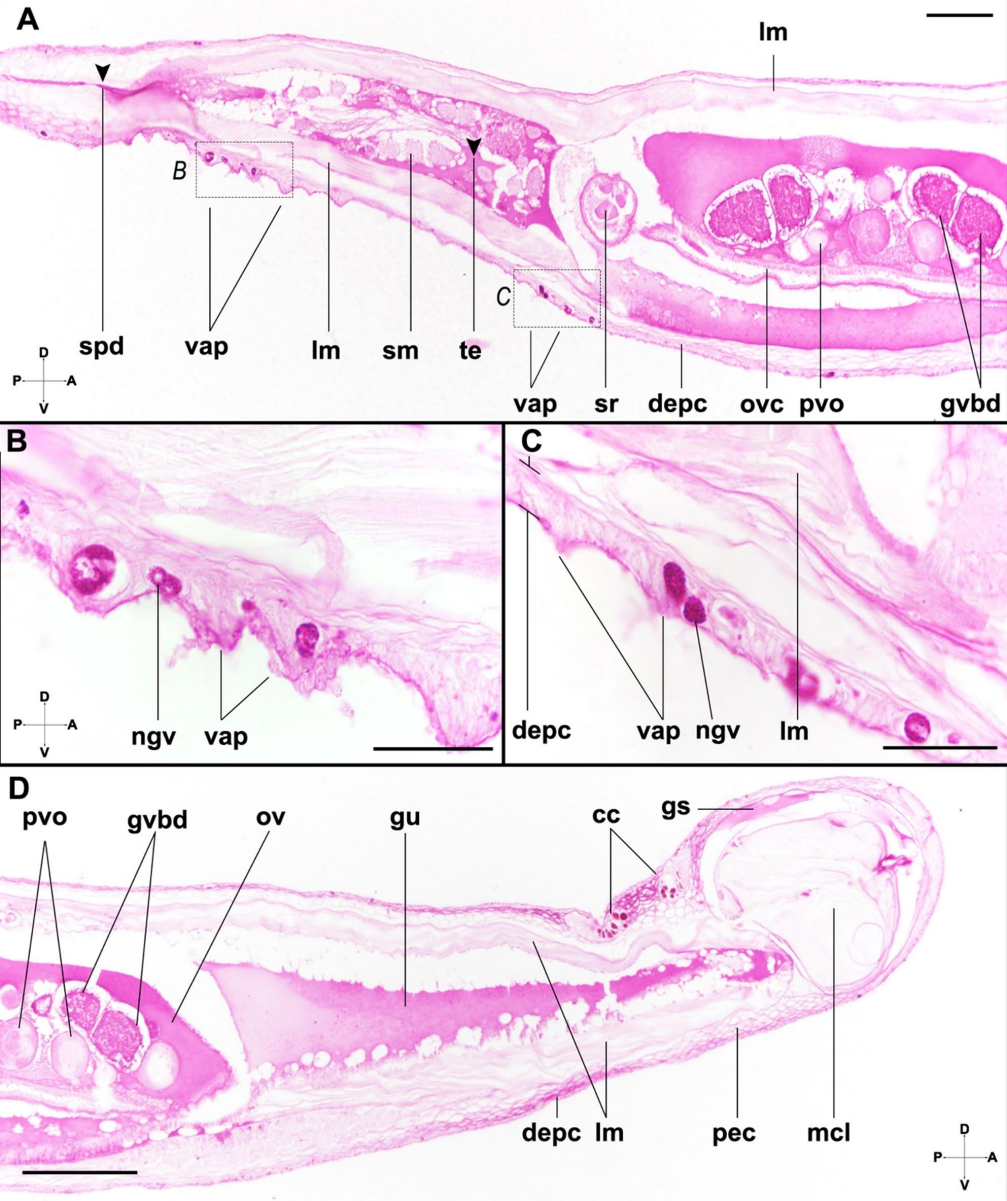
Organization of neutral carbohydrates detected by PAS staining (magenta) in the adult *Spadella cephaloptera*. **(A)** Sagittal section of the mid-posterior region of the body highlighting staining (arrowheads) in the tail, the sperm duct (spd), and testis (te). **(B)** Clusters of ventral adhesive papillae (vap) from the most outer layer of epidermis in the tail (referenced in A) containing strongly stained neutral glycan vesicles (ngv). **(C)** Adhesive cells located below the posterior septum (noted in A), displaying dense vesicles (equivalent to those observed in **B**.). **(D)** Anterior region of the body of *S. cephaloptera* in which the grasping spine (gs) exhibits carbohydrate staining and no groups of vesicles are detected in the distal (depc) or proximal epidermal cells (pec). Additional abbreviations: cc, corona ciliata; gs, grasping spine; gvbd, germinal vesicle breakdown; gu, gut; lm, longitudinal muscles; mcl, lateral muscle; ov, ovary; ovc, oviducal complex; pvo, previtellogenic oocyte; sm, spermatogonial masses; sr, seminal receptacles. Scale bars: A = 100 μm; B, C = 50 μm; D = 200 μm

The neutral carbohydrates detected by the PAS reaction are present in different body regions of *S. cephaloptera* (Fig. 4). In the tail region, staining appears in the sperm duct and includes the testis in the tail coelomic cavities (Fig. 4A). Strongly stained vesicle-like structures are concentrated along the ventral surface of the anterior tail region, near the trunk-tail septum. The glycoconjugates identified in this region appeared to co-localize with the cell clusters forming the ventral adhesive papillae (Fig. 4B–C). These neutral carbohydrates in the distal epidermal layer showed a posteriorly directed distribution, extending toward the transition zone between the trunk and tail. A strong PAS+ signal was also observed in the dorsally-located corona ciliata and in the grasping spines (Fig. 4D). Notably, in the anterior region of the epidermis, no staining could match the one observed in ventral adhesive papillae in size and density (Fig. 4A, D). Treatment with α-amylase abolished the intense vesicle-like staining in the ventral adhesive cells, leaving faint staining in the cell bodies, indicating that these structures contained glycogen. PAS-positive staining persisted after the α-amylase treatment in the spermatogonial masses and the gut, likely representing different glycoproteins and mucins (Suppl. Figure 1).

### Ultrastructure of the developing adhesive system

**Fig. 5:**
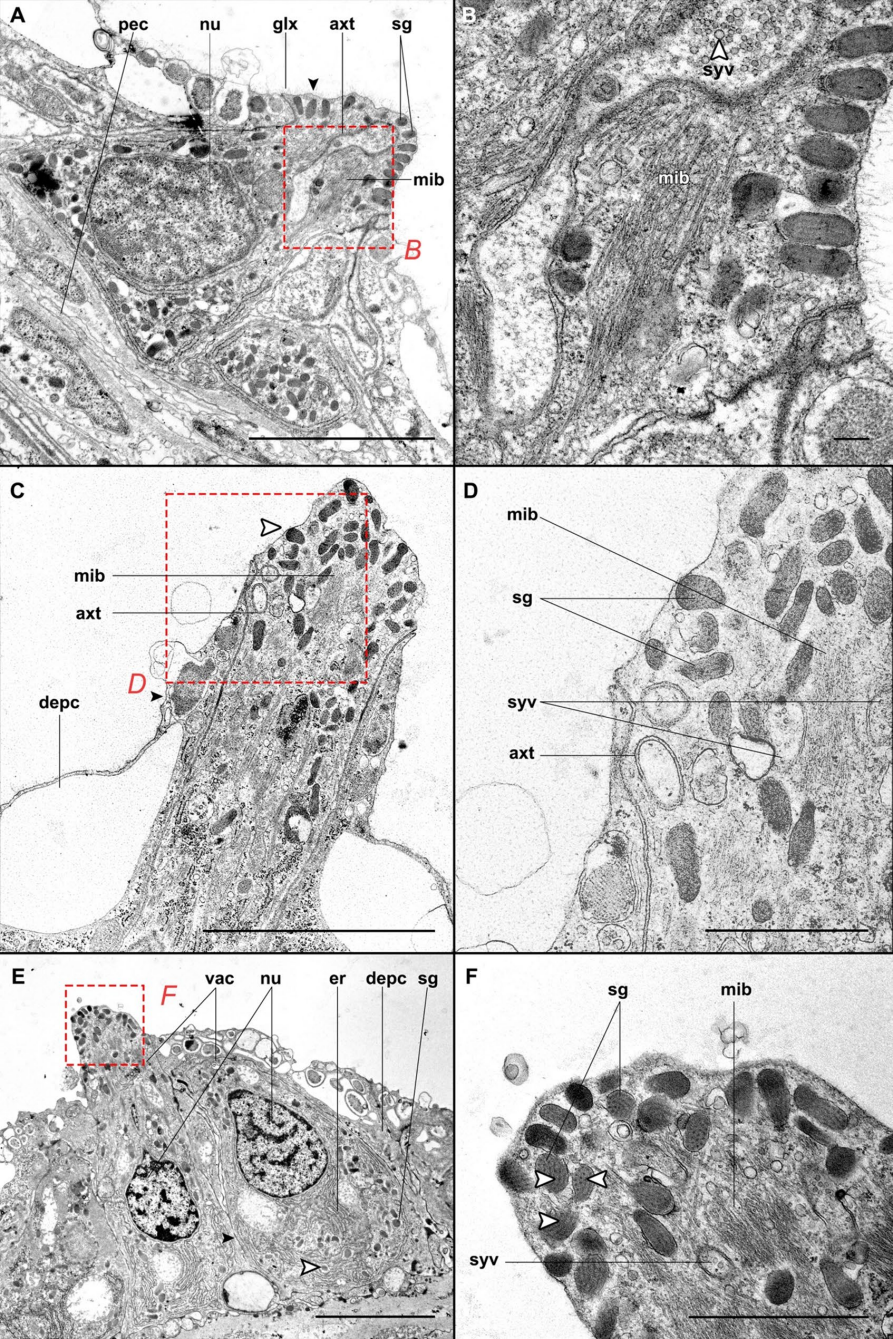
Transmission electron microscopy images of the ventral adhesive papillae during the ontogeny of *Spadella cephaloptera*. **A.** Overview of the ventral adhesive papillae at 24 hph, characterized by the pyramidal elongated shape with a large nucleus in the basal region, placed above the proximal epidermis layer (pec) and exhibiting axonal terminals (axt) inside. **B**. Detail of the apical region of the adhesive cell (referenced in A), where secretion granules (sg) accumulate close to the membrane. Synaptic vesicles (syv) can be seen (arrowhead) within the axonal terminal. **(C)** The ventral adhesive cell at 5 dph exhibits similar features to those at 24 hph (A), and is surrounded by distal epidermis cells (depc), but notably more elongated. The cell also contains electron-dense granules accumulated in the apical region (white arrowhead) and is surrounded by epidermal cells that likely are releasing material to the exterior (black arrowhead). **(D)** Close-up of the adhesive cell apical region (referenced in C), where the microtubules (mib) and double-membrane axonal terminals are present. **(E)** Overview of two elongated ventral adhesive cells (vac) in the adult of *S. cephaloptera* being in physical contact (arrowhead) and embedded in the distal epidermis region. Note how the secretion granules are found in the basal region next to the endoplasmic reticulum (er) (white arrowheads). **(F)** Close-up of the most apical part of the adhesive cell (noted in E), in which the fiber-like content inside the secretion granules is evident (arrowheads). Additional abbreviations: nu, nucleus. Scale bars: A, C, E = 5 μm; B = 200 nm; D, F = 1.25 μm

We examined the ultrastructure of the adhesive cells using transmission electron microscopy (TEM) to characterize the subcellular structures likely involved in attachment. At 24 h post-hatching (24 hph), specimens of *S. cephaloptera* possess ventral adhesive cells of pyramidal shape (Fig. 5A). These cells are embedded in the apical region of the multilayered epidermis and protrude as papillae. In the basal region of the adhesive cell, a nucleus occupies a significant portion of the cell. In the cytoplasm, there is an abundance of filaments that are about the size of microtubules (Fig. 5A, B) [42]. Numerous electron-dense granules are present in the adhesive cell bodies and enriched towards the apical region, where they are presumably secreted (Fig. 5A, B). The presence of ribosomes near these granules suggests that active protein synthesis supports the granule formation (Suppl. Figure 2 A). The surrounding epidermal cells envelop most of the cell body of the adhesive cells, leaving a small uncovered area relative to the whole cell size. At this stage, all adhesive cells are discretely distributed, with no evidence of physical contact between them (Fig. 5A).

At 5 days post-hatching (5 dph), the size of each adhesive cell of *S. cephaloptera* increases about 1.3 times compared to 24 hph (Fig. 5C). The electron dense granules, described for 24 hph specimens, are similarly clustered in the apical-most region of the adhesive cells at 5 dph (Fig. 5C). In this regard, the adhesive cells of 5 dph specimens showed evidence of exocytosis (Suppl. Figure 2B). Moreover, these secretory granules revealed parallel, strip-like structures (Fig. 5D). No evidence of contact between different adhesive cells was observed at this developmental stage.

In adult *S. cephaloptera*, abundant electron-dense secretory granules of multiple sizes are visible in the distal-most layer of the epidermis, suggesting high secretory activity (Suppl. Figure 2 C). The membranes of individual adhesive cells are in physical contact with each other, resulting in clusters of at least two cells (Fig. 5E). The fiber-like structures, already observed in the electron-dense secretory granules of 5 dph old specimens, are still present in adults (Fig. 5F). Consistent with previous results in adults, synaptic vesicles were identified in all developmental stages examined (Fig. 5B, D, F) [12].

Distribution of the adhesive cells during ontogeny.

**Fig. 6:**
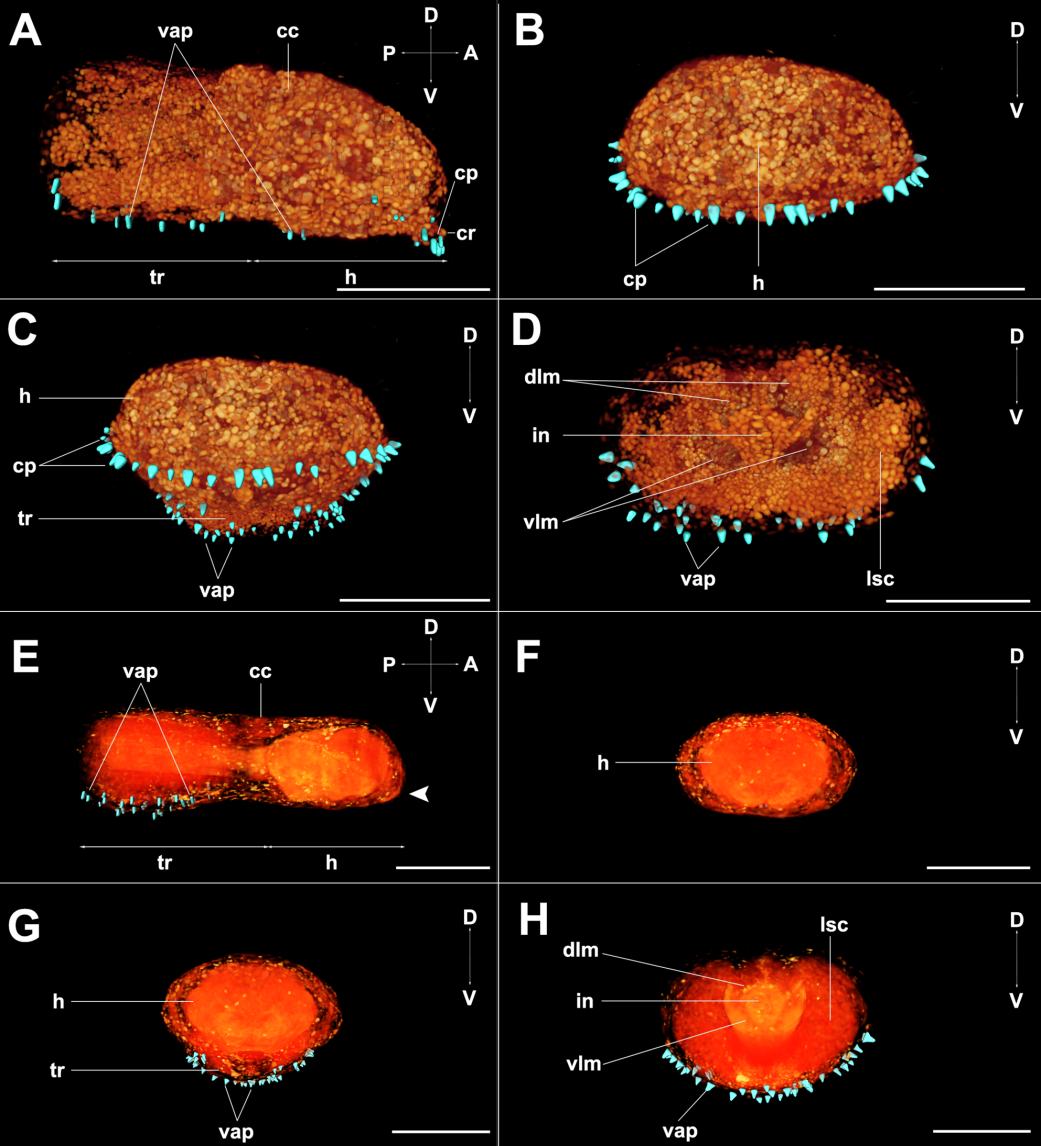
3D reconstructions of the anterior part of the body (trunk and head) of *Spadella cephaloptera* highlighting the distribution of the ventral and cephalic adhesive papillae. Volume rendering (orange) and manually segmented papillae (cyan). **(A)** Lateral view in a 24 hph individual, where ventral adhesive cells (vap) are distributed in the ventral head (h) and trunk (tr) regions. Additional adhesive cells are present in the cephalic rim (cr). **(B)** Front view of the anterior part of the head at 24 hph, where cephalic papillae (cp) are distributed along the cephalic rim. **(C)** Tilted front view of the anterior region of the hatchling at 24 hph, where the distribution of the cells can encompass the ventral surface. **(D)** Transversal section of the trunk of the hatchling at 24 hph, showing the location of the ventral adhesive papillae on the distal epidermis with respect to internal systems such as the lateral somata clusters (lsc) and the longitudinal muscles. **(E)** Lateral view at 5 dph showing the absence of the cephalic rim (arrowhead) at this stage in the anterior part of the head. **(F)** Front view of the anterior region of the head at 5 dph devoid of cephalic papillae. **(G)** Tilted front view of the anterior region of the body, showing how the ventral adhesive cells in the trunk follow a similar distribution in the frontal plane to the one at 24 hph. **(H)** Transversal section of the trunk where ventral adhesive cells form a multiple adhesive system as part of the epidermis. Additional abbreviations: cc, corona ciliata; in, intestine; dlm, dorsal longitudinal muscle; vlm, ventral longitudinal muscle. Scale bars: 100 μm

Using 3D reconstructions, we analyzed the distribution of adhesive cells in *S. cephaloptera* and found stage-specific patterns. At 24 hph, adhesive cells are strongly enriched along the cephalic rim, a semi-circular protruding structure in the antero-ventral half of the body (Fig. 6A-C). In the neck region, which connects the head to the trunk, there are few adhesive cells which become more abundant toward the posterior region of the specimen (Fig. 6C). In the ventral mid-trunk region, adhesive cells are not clustered in specific body regions, but rather form a multiple-point adhesive system (Fig. 6D). In 5 dph old specimens, the anterior cephalic rim and the associated adhesive cells which protrude as papillae are absent (Fig. 6E-G). Instead, the adhesive cells display an increased density in the trunk region (Fig. 6E, H).

### Immunohistochemical characterization

**Fig. 7:**
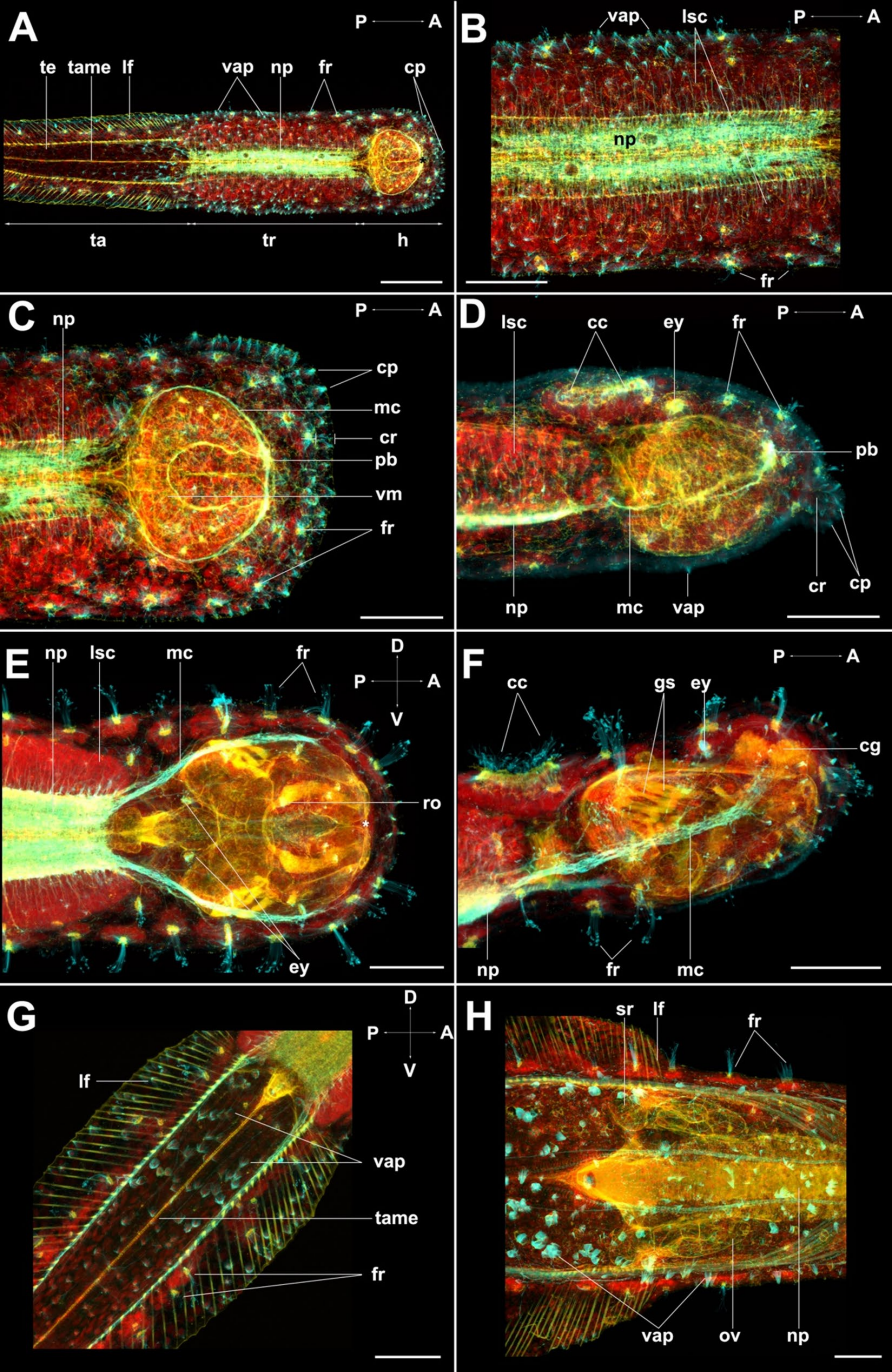
Distribution of acetylated microtubules (cyan), actin filaments (yellow), and cell nuclei (red) during the ontogeny of *Spadella cephaloptera*. **A.** Overview of labelled structures at 24 hph, which include the fence receptors (fr), the ventrally distributed adhesive papillae (vap), and the cephalic papillae (cp.). **B.** Maximum projection of the trunk (tr) region of 24 hph hatchling showing microtubules extending from the ventral neuropil (np), which is surrounded by the lateral somata clusters (lsc). **C.** Detail of the anterior part of the head where microtubules in the cephalic papillae distribute on the cephalic rim (cr). **D**. Side view of the hatchling, which shows the microtubule processes in the anterior end of the head. Notably, this structure is not directly connected to the primordial brain (pb). From this view, it can be seen how the fence receptors and the cephalic papillae connect to the intraepidermal plexus. **(E)** Maximum projection of the anterior part of the head of a 5 dph specimen, showing intense signal from acetylated microtubules associated with structures of the nervous system, such as the ventral neuropil, and the main connectives (mc). Notably, the distal epidermis is populated with multiple fence receptors, which now are in the region of the abolished cephalic rim. **(F)** Side view of the head at 5 dph showing how acetylated microtubules are associated with multiple sensory organs. **(G)** Detail of the tail of a 5 dph specimen showing the distribution of ventral adhesive papillae in the tail, which involves the lateral fin (lf). **(H)** Close up of the transition zone between the trunk and tail in the adult of *S. cephaloptera*. Additional abbreviations: cc, corona ciliata; cg, cerebral ganglion; ey, eye; gs, grasping spine; h, head; ov, ovary; ro, retrocerebral organ; sr, seminal receptacle; tame, tail mesentery; vm, vestibular muscles. Scale bars: A = 100 μm; B-H = 50 μm

To examine potential neural connections to adhesive cells, we fluorescently labeled acetylated α-tubulin and stained F-actin with phalloidin to visualize the cytoskeletal organization of the adhesive cells. Strong immunoreactivity was detected in the adhesive cells protruding as papillae (Fig. 7A-D, G-H). In hatchlings (24 hph), adhesive cells of the anterior-most head region including the cephalic rim exhibit acetylated microtubules (Fig. 7A), a pattern that is also present along the trunk extending to the level of the posterior septum (Fig. 7A). At the magnification examined, discrete nerve bundles extending directly from the VNC to individual adhesive cells could not be resolved (Fig. 7B). Moreover, the forming cephalic ganglia showed no visible processes connecting to cephalic rim adhesive cells (Fig. 7C, D). Notably, beneath the adhesive cells, a subjacent nervous plexus is located as revealed by anti-acetylated α-tubulin in the epidermis (Fig. 7D, Suppl. Figure 3). Individual adhesive cells feature acetylated microtubule populations, but the spatial relationship between these microtubules and the neural plexus cannot be resolved (Suppl. Figure 3). Regions of intense F-actin signal, co-localizing with acetylated α-tubulin, were observed in the epidermis (Fig. 7A-D). The signal from actin filaments within the adhesive cells was weaker throughout development than that observed in muscle-related structures, suggesting that the cells rely on a different mechanism for structural support (Fig. 7).

At 5 dph, the specimens are characterized by an abundance of cephalic ciliary receptors as evidenced by acetylated α-tubulin and F-actin processes (Fig. 7E-F). The acetylated microtubules related to the adhesive cells of the cephalic rim were not detected. Instead, short ciliary receptors are present in this region (Fig. 7E). At 5 dph, adhesive cells are present in clusters more posteriorly in the ventral region of the tail and as individual cells on the lateral fin surfaces (Fig. 7G). In adults, acetylated microtubules associated with the adhesive cells are arranged in clusters, predominantly located in the anterior tail region (Fig. 7H).

### Lectin affinity

**Fig. 8:**
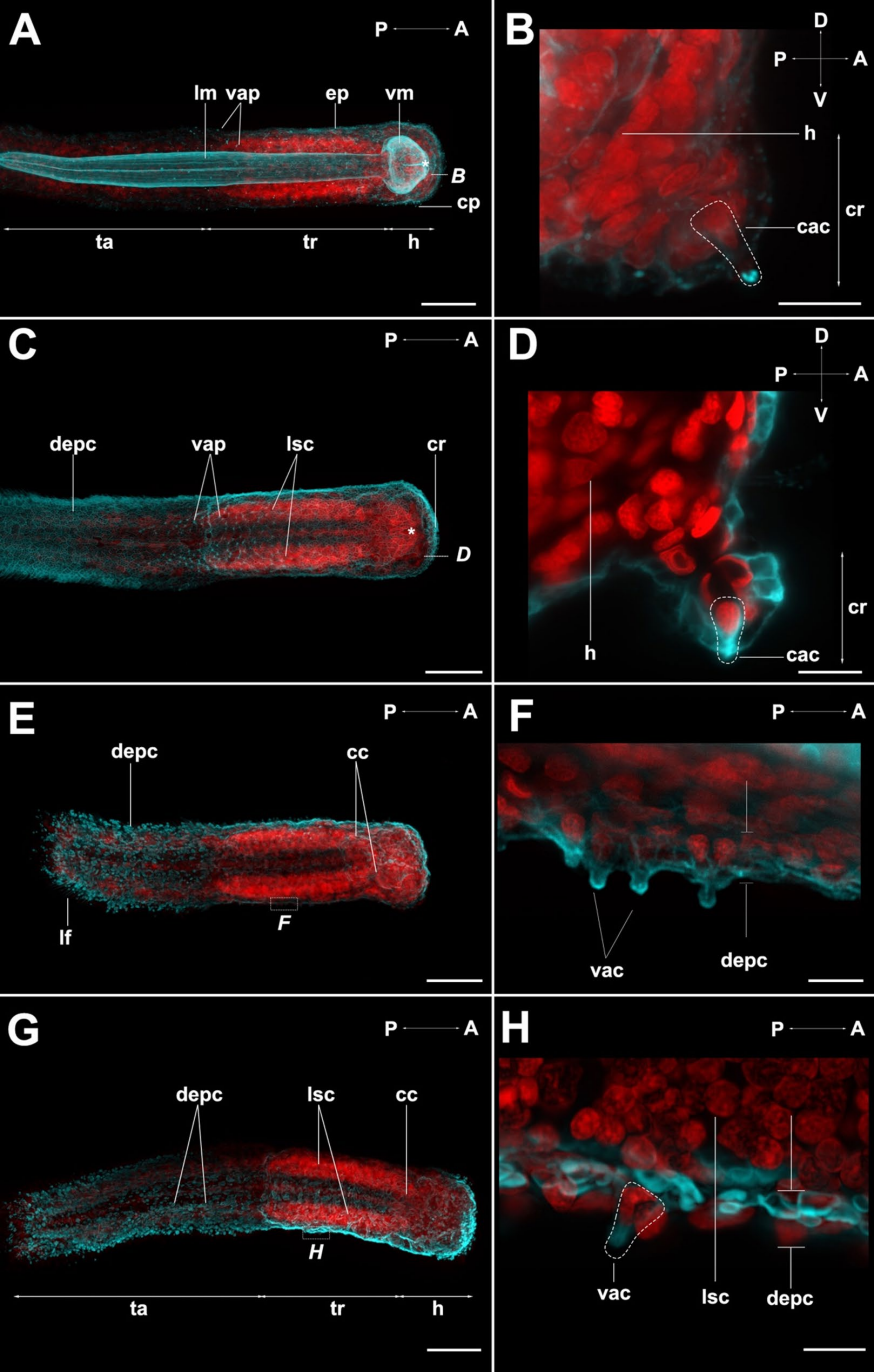
Distribution of glycans detected by lectin-binding affinity (PNA, Con A, SBA and UEA) on hatchlings (24 hph) of *Spadella cephaloptera*. The signal of the lectin histochemistry is colored with cyan, and the nuclei with red. **(A)** Peanut agglutinin (PNA) related signal labelled the longitudinal muscles (lm) and vestibular muscles (vm) and faintly the epidermis (ep). **(B)** Detail of the cephalic rim (cr) in the anterior head (h) region (referenced in A), where PNA-signal strongly labels the apical region of the cephalic adhesive cell (cac) (dotted line). **(C)** Concanavalin A (Con A) bound to glycans in the cell membranes in the distal region of the epidermis (depc). **(D)** Close-up of the cephalic rim (cr) (referenced in C), where glycans detected by Con A are present in the cephalic adhesive cells (dotted line) and surrounding epidermal cells. **(E)** Location of the glycans for which *Phaseolus vulgaris* Erythroagglutinin (Pha-E) has binding affinity. The lectin bound preferentially to the surface of the hatchling’s epidermis. **(F)** Magnified trunk region (referenced in E) showing multiple ventral adhesive cells (vac) labelled by Pha-E, with higher signal intensity in the apical region of the cell. **(G)** Overview of the glycans detected by *P. vulgaris* leucoagglutinin (Pha-L), which are distributed through the epidermis. **(H)** Detail of trunk (referenced in G) showing distinct labeling in the distal epidermal cells (depc) and the ventral adhesive cells (vap). Additional abbreviations: cc, corona ciliata; lf, lateral fin; lsc, lateral somata cluster; ptz, posterior transition zone; ta, tail; tr, trunk; vap, ventral adhesive papillae. Scale bars: A, C, E, G = 100 μm; B, D = 10 μm; F = 50 μm; H = 25 μm

Lectin histochemistry in 24 hph specimens showed that the adhesive cells feature a distinct glycosylation profile compared to the general epidermis. The adhesive cells are characterized by strong enrichment in galactosylated and mannosylated moieties. Among the lectins tested, PNA labeled the adhesive cells strongly, with signal strongest in the apical region (Fig. 8A, B), suggesting a higher concentration of glycans in the region enriched in secretory granules (Fig. 5A, C, E). The lectins Con A and Pha-E labelled the adhesive cell bodies and membranes (Fig. 8C-F). Pha-L showed a broader distribution, with labeling in the distal and proximal epidermal layers and within the cell bodies of the adhesive cells (Fig. 8G-H), but with lower intensity compared to PNA, Con A, and PhaE.

SBA revealed a granule-like pattern (punctate labeling) observed with low abundance in the adhesive cells (including those in the cephalic rim). In contrast, adhesive cells do not show (or only faintly) fucosylated glycans detected by UEA. The latter are, however, present in the proximal and distal epidermis (Suppl. Figure 4 C-D). Other lectins did not label adhesive cells, but did label the developing neuromuscular and sensory systems. Succinated WGA showed strong affinity for glycans present in the ciliary receptors, particularly those in the fin (Suppl. Figure 4E), but showed no specific enrichment in the adhesive cells (Suppl. Figure 4 F). Muscle-related signal was detected when using PNA, in the longitudinal muscles and the vestibular region (Fig. 8A), while SBA labeled the forming lateral plates, the eyes and the developing mouth (Suppl. Figure 4 A).

## Discussion

### Glycan distribution in reproductive systems

**Fig. 9:**
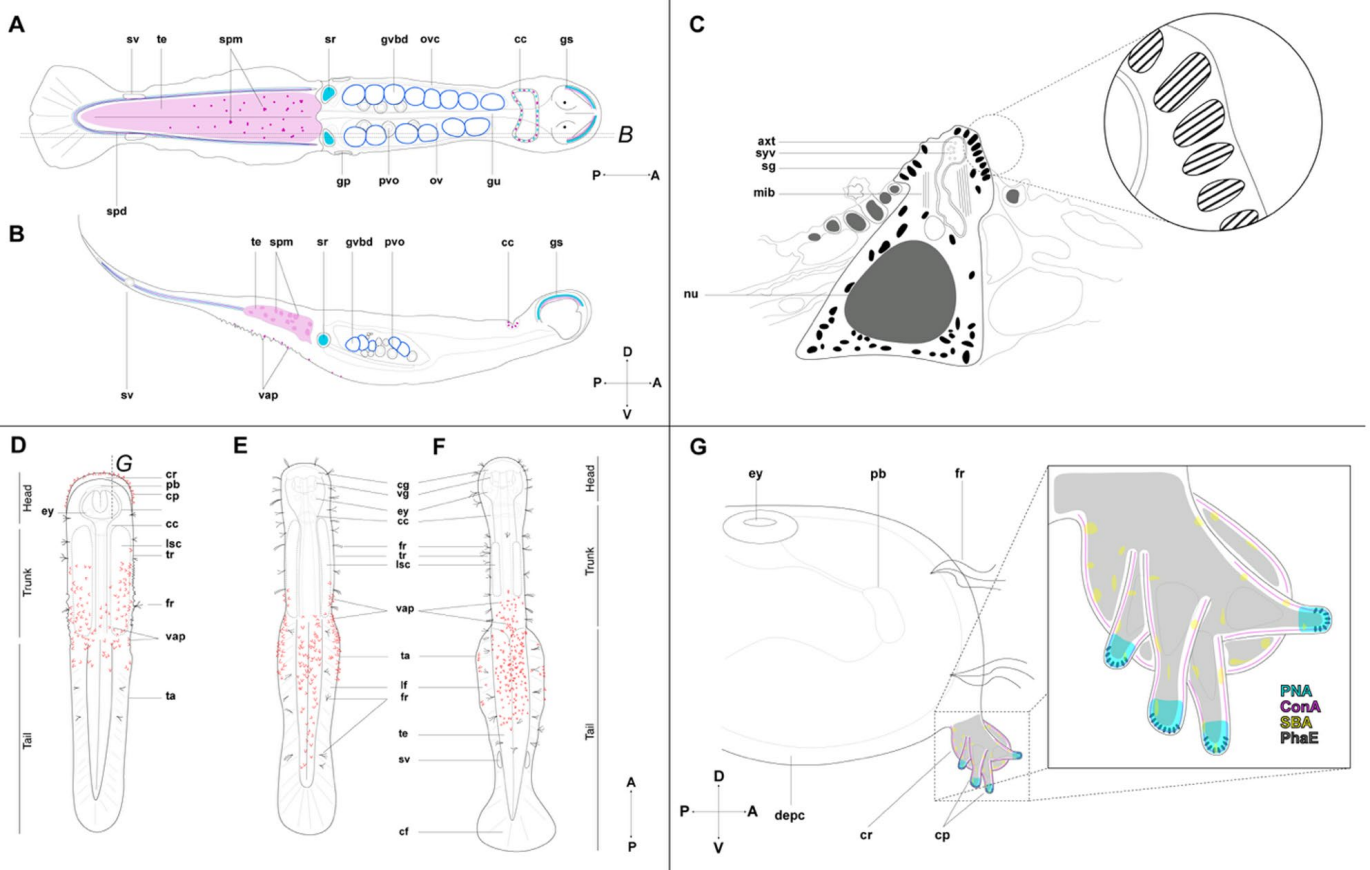
Schematic summary of the findings from the histological and ultrastructural analyses on *Spadella cephaloptera.* **(A)** Distribution of glycans in the reproductive system. The location of Alcian blue (pH = 2.5)^+^ polysaccharides (dark blue) includes the sperm duct (spd) and oocytes after germinal vesicle breakdown (gvbd). Alcian blue (pH = 1.0)^+^ polysaccharides (light blue) line the sperm duct, and the seminal receptacles (sr). In the head region, the dorsal grasping spines (gs) and the corona ciliata (cc) are stained. Neutral glycans (purple) are observed within the sperm duct and in the testis (te). **(B)** Sagittal section (referenced in A), showing PAS+ staining in ventral vesicle-like structures. **(C)** Representation of the ultrastructure of one adhesive papilla (pyramid-like cell). These cells have secretion granules (sg), which feature fiber-like content. **(D)** Ventral view of a 24 hph specimen, characterized by the presence of the cephalic rim (cr) in the anterior part of the head. This structure is enriched in cephalic papillae (cp.) (red). Additional ventral adhesive papillae are in the trunk. **(E)** Overview of the ventral surface at 5 dph, where the cephalic rim is abolished, and adhesive cells are shifted posteriorly. **(F)** Representation of the ventral view of the adult, showing the distribution of the adhesive cells. **(G)** Sagittal section of the head of a specimen at 24 hph (referenced in D), where the lectins labeled moieties: PNA (cyan), Con A (magenta), SBA (yellow), PhaE (gray). Additional abbreviations: axt, axonal terminals; cf., caudal fin; cg, cerebral ganglion; depc, distal epidermis cells; ey, eye; fr, fence receptors; gp, female gonopore; lf, lateral fin; lsc, lateral somata cluster; nu, nucleus; ov, ovary; ovc, oviducal complex; pb, primordial brain; pvo, previtellogenic oocyte; spm, spermatogonial masses; sv, seminal vesicles; syv, synaptic vesicles; vap, ventral adhesive papillae; vg, vestibular ganglion

Carbohydrates play essential roles in metabolism, reproduction, structure formation, and attachment in various marine invertebrates [28, 43–47]. In the protandric hermaphrodite *S. cephaloptera*, Alcian Blue staining (pH = 2.5) revealed sulfated and carboxylated polysaccharides in the ciliated sperm duct but not in the seminal vesicles (Figs. 2, 3 and 9A-B) [48]. Since sperm cells are concentrated in the seminal vesicles, the polysaccharides are unlikely to be sperm-borne. Instead, they likely represent epithelial secretions, consistent with TEM evidence of secretory granules in the duct epithelium [12]. The presence of glycans in these secretions may affect properties of the seminal fluid, such as viscosity or pH, which are relevant for sperm migration towards the gonopore.

Moreover, the distribution of carboxylated and sulfated polysaccharides showed that these two types of mucosubstances are simultaneously distributed in the sperm duct of *S. cephaloptera* (Figs. 2C, 3A-B and 9A-B). Both polysaccharide types may co-occur, or a single polysaccharide with mixed charge (e.g., chondroitin sulfate) may be present [49, 50]. Chemical characterization would be necessary to disentangle the identity of the individual mucosubstances.

The distribution of neutral polysaccharides visualized by the PAS^+^ reaction in *S. cephaloptera* was localized within the sperm ducts in the tail region (Figs. 4A and 9A-B). Considering this observation, the neutral glycans observed in the vas deferens of *S. cephaloptera* could also be part of an epithelial secretion, as suggested for the alcianophilic content of this cavity. In *S. cephaloptera*, additional staining is visible in the coelomic cavities of the tail (Fig. 4A), which may correspond to glycogen, as the staining is not observed when α-amylase treatment is applied (Suppl. Figure 1). The detected glycogen likely serves as metabolic fuel for the energetic requirements of the spermatozoa during the fertilization process, an evolutionarily conserved mechanism across marine invertebrates [51].

Additional roles of mucopolysaccharides in marine invertebrates include mediating recognition between oocytes and sperm cells, which are essential for species-specific fertilization [52, 53]. In the case of *S. cephaloptera*, there are reports of a jelly coat being present from the vitellogenic phase of the largest oocytes that will be involved in fertilization [12, 31]. Chaetognaths are characterized by a unique fertilization mechanism that excludes sperm-jelly coat interactions [31, 54]. In this view, while surface carbohydrates often mediate gamete recognition in sea urchins and tunicates, the reproductive strategy of chaetognaths suggests a different functional role [27, 53, 55]. The carboxylated (but not sulfated) nature of the jelly coat by Alcian Blue (pH = 2.5) (Figs. 2E-F and 9A-B) contrasts with fucosylated sulfated polysaccharides in the surface of sea urchin egg coat [53, 56]. This, however, appears to be consistent with the chaetognath fertilization mechanism, in which the sperm cell reaches the oocyte through a fertilization canal formed by accessory fertilization cells [31, 54]. The carboxylated glycans may instead be involved in the protection of oocytes, as it has been observed for non-sulfated chondroitin in *Caenorhabditis elegans* [57, 58]. So far, there are few reports of non-sulfated mucopolysaccharides in marine invertebrates [59], and further chemical identification is necessary to clarify the role of these glycans associated with chaetognath oocytes.

### Glycan distribution in adhesive systems

The presence of PAS^+^ vesicles in the ventral adhesive cells of *S. cephaloptera* suggests secretion of neutral hexose mucosubstances (c.f [40]) (Figures 4A-B and 9B). Since the α-amylase treatment abolished this staining, these vesicles probably contain glycogen, which is also present in temporary adhesive systems of cnidarians and cephalopods (Suppl. Figure 1) [60–63]. Such glycans could contribute to adhesion through electrostatic interactions between their polar groups and the contacting surface [2, 64].

### Ultrastructure of the adhesive cells

The ultrastructure of the adhesive cells of *S. cephaloptera* reflects the conformational changes the adhesive system undergoes during ontogeny. From the moment of hatching up to 5 dph, individuals of *S. cephaloptera* exhibit discrete, individual adhesive cells embedded in the distal layer of the epidermis (Figs. 5A and 9C) [12]. In contrast, in late juveniles and adults of *S. cephaloptera*, adhesive cells are organized in multicellular clusters with cells in direct membrane contact (Fig. 5E). This ontogenetic transition from individual cells to clustered adhesive cells aligns with the behavioral shift from non-feeding early hatchlings to active predators in early stages.

Electron-dense secretion granules show a polarized apical distribution in the adhesive cells of *S. cephaloptera* (Figs. 5A, C and E and 9C) and the adhesive appendages of the closely related *Paraspadella gotoi* [65], which may enable rapid release upon specific stimuli. In adult specimens of *S. cephaloptera*, we observed that these electron-dense granules can presumably fuse with the cell membrane and release their content (Suppl. Figure 2B). Consequently, the adhesive cells of *S. cephaloptera* behave much like exocrine glands that undergo regulated exocytosis [12].

Notably, our TEM analysis revealed, for the first time, the fibrous nature of the secretion granule content in the adhesive cells of *S. cephaloptera* (Figs. 5D and F and 9C). Previous research on the ultrastructure of the adhesive appendages of *P. gotoi* showed comparable secretory granules with two distinct electron-dense regions forming parallel strands [65]. The formation of such arrangements has been linked to the presence of functional amyloid proteins in marine adhesive systems [66, 67], in which β-sheet conformations provide stability of the extracellular matrix [68]. A recent study highlighted the prevalence of epidermal growth factor (EGF)-like domains in marine adhesive proteins, which form β-sheet regions that may confer stability to those proteins [3]. In the case of *S. cephaloptera*, transcriptomic cell-type analyses, together with thioflavin T immunohistochemistry, may help clarify the molecular identity of the fibrous content in the secretion granules of the adhesive cells.

Dual-gland systems for temporary adhesion, including dedicated cells for adhesion and detachment, respectively, are present in a variety of distantly related organisms. In *S. cephaloptera*, we did not find evidence for specialized detachment cells; instead, the adhesive cells are surrounded by regular epidermal cells (Figs. 5A, C and E and 9C). Ultrastructural evidence of supercontracting muscles associated with the adhesive appendages of *P. gotoi* indicates an adaptation for high-velocity movements, specifically swings and jumps [65]. Such muscular systems are present in different spadellids [12, 65]. Considering the behavior of *S. cephaloptera*, which involves quick flicks rather than continuous swimming [17], a fast-reaction detachment mechanism is required. Therefore, supercontractor muscles likely mediate detachment in *S. cephaloptera*, as suggested in other benthic chaetognaths [65].

### Stage-specific spatial distribution of adhesive cells

The spatial distribution of adhesive cells shifts during the ontogeny of *S. cephaloptera*, matching behavioral and physiological transitions (Figs. 6 and 9D-F). In hatchlings (24 hph), a functional mouth, digestive tract, and anus are absent, which prevents feeding [12, 14]. During this period, the hatchlings constantly attach with the anterior body region to the substrate, likely using their adhesive cells that protrude as adhesive papillae on the cephalic rim (Figs. 6A-C and 9D). The ventral adhesive papillae of the trunk establish a multiple point adhesive system as described for other metazoans (Figs. 6A and E and 9D-F) [7]. The lack of cephalic adhesive cells and the increased density of those cells in the trunk and tail regions in 5 dph individuals correlate with the formation of the alimentary canal and anus, when yolk reserves deplete and digestion commences [12, 17] (Figs. 6E-G and 9E). During this time window, the rearrangement of adhesive cells provides stable anchorage needed for active prey capture and to withstand water currents. In *Spadella schizoptera*, cephalic papillae persist up to the fourth day after hatching, followed by a similar re-distribution pattern observed in *S. cephaloptera* [69, present study]. This suggests that this ontogenetic distribution represents a key adaptation in benthic chaetognaths to balance survival during early stages with feeding requirements during subsequent development.

### Neural connections and cytoskeletal distribution

Previous research on the distribution of acetylated α-tubulin in *S. cephaloptera* focused on nervous system anatomy, however, ventral adhesive cells were also labeled [18]. In line with this study, the distribution pattern of acetylated microtubule processes of different mechanosensory receptors (ciliary fence and tuft receptors) (Fig. 7C-F) has been associated with innervations of the intra- and basiepidermal plexus. These epidermal networks are part of the peripheral nervous system and are considered an autapomorphy of chaetognaths. They communicate input from the different ciliary receptors distributed throughout the body of *S. cephaloptera* to the VNC [12, 18].

In the present study, we investigated the distribution patterns of acetylated microtubules searching for processes derived from neuronal ganglia that could suggest associations with the adhesive cells (Fig. 7). In this aspect, we did not resolve distinct acetylated α-tubulin nerve-related processes forming a pattern that indicates direct association between the ventral nerve center (VNC) (Fig. 7B) or the cephalic ganglia (Fig. 7C) to individual adhesive cells. Instead, multiple acetylated microtubule bundles inside the cell bodies of the adhesive cells projected toward the epidermis, which hosts the intra- and basiepidermal neuronal plexus. Given these observations and the presence of synaptic vesicles in the cell bodies of the adhesive cells observed through ontogeny (Fig. 5B, D, F) [12], adhesive cells may participate in a neurosecretion mechanism that releases on demand the secretion granules that contain agents potentially involved in the attachment process (Suppl. Figure 2B). It should be noted that acetylated α-tubulin, commonly used to detect microtubules, is not restricted to neurons. Therefore, TEM data showing vesicles surrounded by membranes (consistent with synaptic vesicles) should receive greater weight for inferring neural associations. The presence of such vesicle-like structures hints towards a relationship between the peripheral nervous system and the attachment process in *S. cephaloptera* (Fig. 5 & Suppl. Figure 2).

### Distribution of lectin-binding glycans in the hatchling stage

Glycans have been demonstrated to be part of different temporary adhesive systems in marine invertebrates as revealed by histological staining experiments (e.g., PAS staining, AB) [60] and immunohistochemistry based on lectin affinity for specific moieties [9–11, 70–73]. These results must be interpreted with caution, as glycans serve cellular processes beyond adhesion. Thus, while the lectin-binding signal confirms their presence, additional functional studies are required to verify their specific role in attachment. PNA, which recognizes Galβ1-3GalNAc epitopes [11], showed affinity to the apical region of the adhesive cells (Figs. 8B and 9G), where TEM revealed a higher concentration of the electron-dense granules (Figs. 5A, C and E and 9G). PNA is known to bind O-linked glycans attached to serine/threonine residues [74]. O-glycosylation increases protein stability while reducing flexibility and can favor protein secretion [75]. The apical concentration of PNA-binding moieties suggests that either the secretion granules contain an O-glycosylated protein or some galactose-containing carbohydrate [76], sufficiently stable when secreted to mediate substrate interactions. High levels of O-glycosylation have been linked to polymeric gel-forming mucins with the capacity to form hydrogels, which would allow for maintaining substrate contact while preserving the viscoelastic properties required for temporary attachment [77].

The lectin Concanavalin A (α-Man/α-Glc-binding lectin) strongly labeled the membranes of the epidermis and the ventral adhesive cells of *S. cephaloptera* (Figs. 8C-D and 9G), similar to the observed pattern in the platyhelminth *Macrostomum lignano* [9]. Furthermore, the presence of Con A-binding glycans is consistent with the presence of N-glycosylated proteins occurring inside the adhesive cells [78]. In other eukaryotes, N-glycosylation limits protein aggregation prone to misfolding [79]. Thus, beyond providing electrostatic degrees of freedom for adhesion, N-glycosylation may prevent the uncontrolled aggregation of the hypothetical protein content of the glue of *S. cephaloptera* before its release.

The lectin PHA-E, which has affinity for complex branched mannose structures, strongly labelled the distal layer of the epidermis (Fig. 8E-F). The signal was also observed in the cell bodies of the ventral adhesive cells, with the brightest signal at the apical region of the cell (Figs. 8F and 9E). Similar distributions occur in tunicate larvae, including *Ciona intestinalis*, where PHA-E binds preferentially to glycans in the tip of the papillary organ and the adhesive plaque [10], suggesting that these complex glycan moieties may represent additional conserved features of secretions in marine temporary adhesive systems.

The lectin SBA labeled both the epidermis and the adhesive cells of *S. cephaloptera*, resembling a granular pattern with reduced specificity and abundance in the adhesive papillae compared to PNA and Con A, respectively (Suppl. Figure 4 A-B, 9G). SBA confirmed O-linked N-acetylgalactosamine in the adhesive disc of *P. lividus* through lectin pull-downs [80], but was absent from the adhesive organ cells of *M. lignano* [9]. On this basis, the presence of N-acetylgalactosamine monosaccharides is less specific to adhesive organs of marine invertebrates than the Galβ1-3GalNAc disaccharide detected by PNA. This observation can be related to the fact that PNA-binding glycans are characteristic of mucins, which are prevalent in marine adhesive systems [5]. Pha-L-binding detected moieties such as Galβ4GlcNAcβ6 (GlcNAcβ2Manα3) Manα3 were observed in the epidermis and the adhesive cells of *S. cephaloptera* (Fig. 8G, H). Comparable results for Pha-L-binding have been reported for the adhesive organ and footprint of the platyhelminth *Minona ileanae*, with moderate intensity in the adhesive disc of the sea urchin *Paracentrotus lividus* [33, 73], but absent in *C. intestinalis*, suggesting that these moieties are also part of less conserved mechanisms [10].

The labelling pattern of UEA, which binds to αFuc-linked to galactose residues, is of low intensity in the ventral adhesive cell bodies of *S. cephaloptera* (Suppl. Figure 4 C-D). UEA-binding glycans are rarely detected in temporary adhesive systems, being detected strongly only in lectin blots from the proteinaceous fraction of the adhesive material of the sea star *Asterias rubens* [2]. Therefore, this type of glycan appears to play a more species-specific role. In the case of succinated WGA, known to bind GlcNAc, no adhesive cell-specific signal is evident in *S. cephaloptera* (Suppl. Figure 4E-F). On this basis, even though the GlcNAc residue is part of the target of multiple lectins, when it is investigated in isolation, there is no evidence that it occurs with higher prevalence in the ventral adhesive cells of *S. cephaloptera*. On the other hand, when additional moieties are present in the glycans, such as those in Galβ3GalNAc (targeted by PNA), and GlcNAcβ4Manα3 (targeted by PHA-E) [11], we found an enrichment in the adhesive cells, suggesting that more complex glycosylation or carbohydrate synthesis processes may be utilized by chaetognath adhesive cells. The presence of (PAS^+^) carbohydrates, the intense PNA binding, and the electron-dense granules in the apical region of adhesive cells argue for a glycan-dependent adhesion process, potentially related to glycoproteins or carbohydrates [3, 81]. Moreover, PNA (O-glycosylation) and Con A (N-glycosylation) labeling suggest that the adhesive system relies on translational modifications that serve multiple functions, such as electrostatic interactions, the prevention of premature aggregation, and the formation of hydrogels, which resemble widespread mechanisms in marine invertebrates [2, 3].

## Conclusion

By integrating histochemistry, lectin labeling, transmission electron microscopy, and cytoskeletal mapping, the present study characterizes the specialized adhesive system of the benthic chaetognath *Spadella cephaloptera*. Our results reveal that adhesive cells undergo an ontogenetic shift from the anterior to the posterior body region, likely correlated with the alimentary and foraging behavior during development. Ultrastructurally, adhesive cells feature apical electron-dense secretion granules with fibrillar content, which likely mediates substrate attachment. In addition, the co-localization of synaptic vesicles and acetylated microtubules, alongside an adjacent intraepidermal plexus, suggests a neurosecretory profile for this system. Our histological analyses demonstrate that glycans are involved not only in adhesion but also in reproductive processes. The presence of neutral mucosubstances and PNA/PhaE-binding motifs suggests that *S. cephaloptera* possesses convergently evolved traits shared with other temporary adhesive systems from marine invertebrates. Nevertheless, the adhesive system of *S. cephaloptera* also exhibits species-specific glycan signatures (O- and N-linked moieties). This study provides a framework for future molecular investigations aimed at identifying the specific proteins, carbohydrates, and hypothetical glycan–protein interactions underlying adhesion in *S. cephaloptera* and placing these mechanisms into a broader evolutionary context.

## Supplementary Information

Below is the link to the electronic supplementary material.

Supplementary Material 1

## Data Availability

All data generated and analyzed during this study are included in this published article and its supplementary information file.
