# Supplementary material for: Glycans in adhesion and fertilization: histochemical and ultrastructural insights from the chaetognath Spadella cephaloptera
Source: Front Zool. 2026 May 9;23:26. doi: 10.1186/s12983-026-00614-5 (PMC13330366; doi:10.1186/s12983-026-00614-5)
Supplement: Supplementary file 1 — Supplementary Material 1 [file 12983_2026_614_MOESM1_ESM.pdf]

Supplementary Figures

# Glycans in adhesion and fertilization: histochemical and ultrastructural insights from the chaetognath *Spadella cephaloptera*

Cristian Camilo Barrera Grijalba[1,3]*, Sabine Thetter-Dürr[2], Julian Bibermair[1,3], Tim Wollesen[1]*

[1] Department of Evolutionary Biology, Faculty of Life Sciences, University of Vienna, Djerassiplatz 1, 1030, Vienna, Austria

[2] Research Support Facilities Imaging Unit CIUS, Faculty of Life Sciences, University of Vienna, Vienna, Austria

[3] Vienna Doctoral School of Ecology and Evolution (VDSEE), University of Vienna, Austria

* To whom correspondence should be addressed:

Tim Wollesen: tim.wollesen@univie.ac.at

Cristian Camilo Barrera Grijalba: cristian.barrera@univie.ac.at

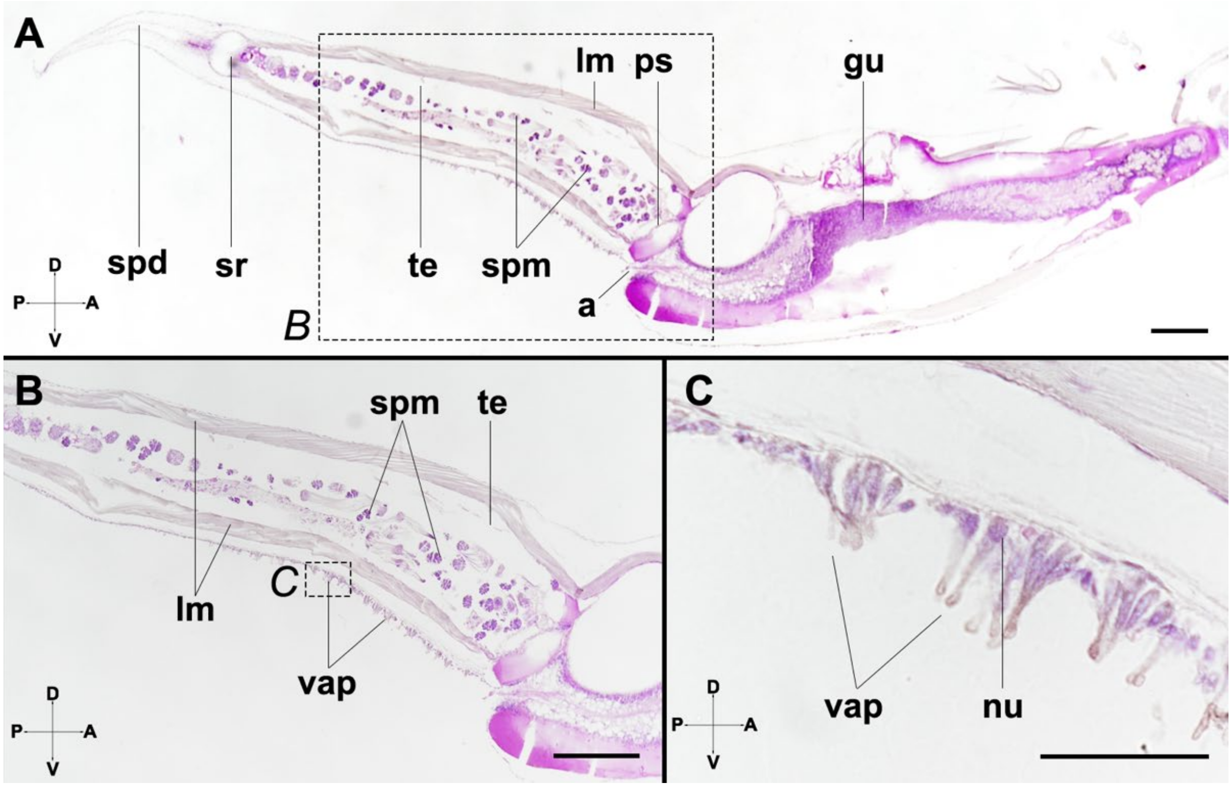


**Supplementary Figure 1.** Negative control for the Periodic Acid Schiff (PAS) reaction. a-amylase digestion before PAS staining in an adult specimen of *Spadella cephaloptera*.

The staining (violet) indicates PAS$^+$ molecules after glycogen digestion and is therefore not included in the discussion. **A.** Sagittal section stained with PAS after a-amylase digestion. In the tail region, the spermatogonial masses (spm) within the testes (te) exhibit staining. Strong staining is visible in the gut (gu) in the trunk region**. B.** Detail of the tail region (referenced in A) showing the absence of staining in the testes. **C.** Close-up of the ventral adhesive papillae (vap), where no PAS$^+$ labelled molecules are present when a-amylase treatment is applied (noted in B). Additional abbreviations: a, anus; lm, longitudinal muscle; nu, nuclei; ps, posterior septum; spd, sperm duct; sr, seminal receptacle. Scale bars: A, B = 200 µm; C = 50 µm.

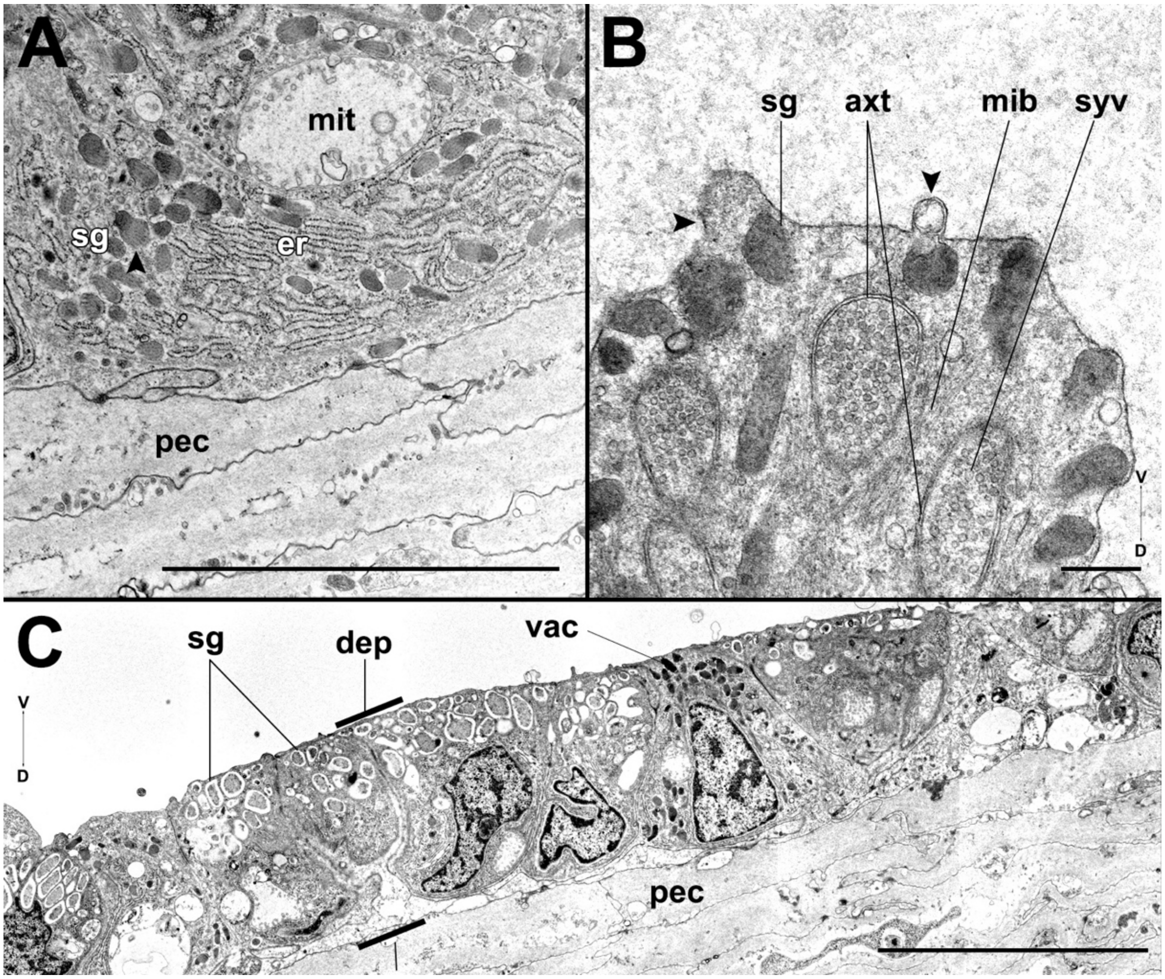


**Supplementary Figure 2.** Ultrastructural features of the adhesive cells of *Spadella cephaloptera*.

**A.** Detail on the origin of the secretion granules (sg) (arrowhead) in the endoplasmic reticulum (er), localized in the basal region of the adhesive cells. **B.** Indication of exocytosis events of the electron-dense granules in the apical region of the adhesive cells (arrowheads) at 5 dph**. C.** Ultrastructure of the distal epidermis (dep), sitting on the proximal epidermis (pec) in the adult chaetognath, where epidermal cells exhibit a high density of secretion granules and surround the ventral adhesive cells (vac)**.** Additional abbreviations: axt, axonal terminal; mib, microtubules; mit, mitochondria; syv, synaptic vesicles. Scale bars: A = 5 μm, B = 500 nm, C = 10 μm.

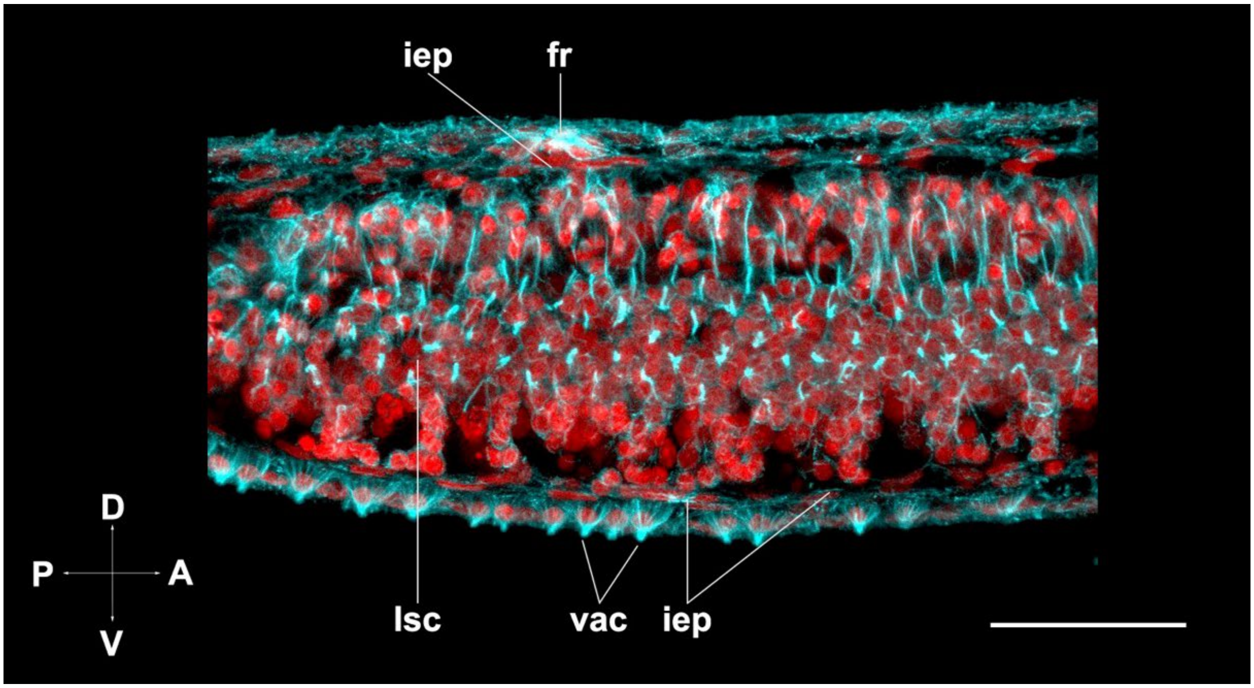


**Supplementary Figure 3**. Distribution of acetylated microtubules in the trunk region of *Spadella cephaloptera* at 24 hph.

From the lateral somata cell clusters (lsc), acetylated microtubules project towards the intraepidermal plexus (iep), which connects to multiple cell types in the surface of the epidermis, such as the fence receptors (fr) and the ventral adhesive cells (vac). Scale bar: 50 μm.

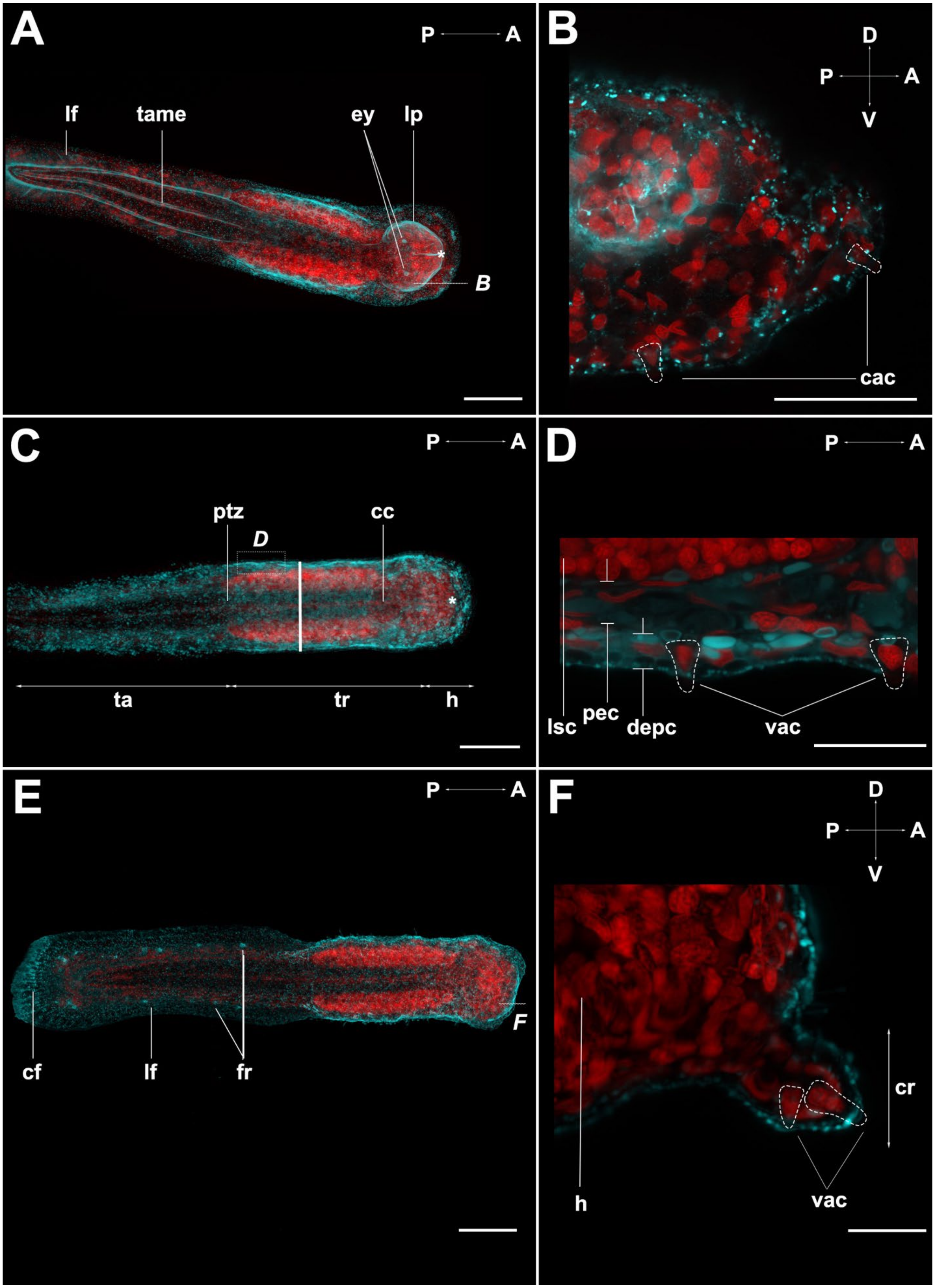

A
P A
lf
tame
ey
lp
B
B
D
P A
V
cac
C
P A
ptz
D
cc
ta
tr
h
D
P A
lsc
pec
depc
vac
E
P A
cf
lf
fr
F
F
D
P A
V
cr
h
vac

**Supplementary Figure 4**. Distribution of glycans detected by lectin histochemistry (SBA, UEA, sWGA) in *Spadella cephaloptera* at 24 hph.

**A.** Overview of the glycans detected by soybean agglutinin (SBA), which lined the tail coelomic cavities and the tail mesentery (tame). In the anterior region, SBA is bound to glycans in the forming lateral plate (lp) and the eyes (ey). **B.** Close-up of the anterior part of the head (h) (referenced in A), where a granular pattern is present in both the epidermis and cephalic papillae (cp). **C.** Maximum projection of the glycans detected by *Ulex Europaeus* agglutinin I (UEA), which are distributed on the epidermis across the body of the hatchling. **D.** Detail of the epidermis in the trunk region (noted in C), showing how UEA labeling involved both proximal (pec) and distal epidermal cells (depc) but showed faint signal in the ventral adhesive papillae (vap). **E.** Maximum projection of the labelled glycans identified by the binding of succinated wheat germ agglutinin (s-WGA). These glycans are in the epidermis and produce distinct labeling signal in the fence receptors (fr) distributed in the tail, and the caudal fin (cf). **F**. Close-up of the anterior part of the head (h) (noted in E), showing a granular pattern in the epidermis with no enrichment of signal in the ventral adhesive papillae (dotted lines). Additional abbreviations: cc, corona ciliata; lf, lateral fin; lsc, lateral somata cluster; ptz, posterior transition zone; ta, tail; tr, trunk. Scale bars: A, C, E = 100 µm; B, D, F = 10 µm.
